# Host Adaptive Evolution of Avian-Origin H3N2 Canine Influenza Virus

**DOI:** 10.3389/fmicb.2021.655228

**Published:** 2021-06-14

**Authors:** Fucheng Guo, Ayan Roy, Ruichen Wang, Jinjin Yang, Zhipeng Zhang, Wen Luo, Xuejuan Shen, Rui-Ai Chen, David M. Irwin, Yongyi Shen

**Affiliations:** ^1^Guangdong Laboratory for Lingnan Modern Agriculture, Guangzhou, China; ^2^Center for Emerging and Zoonotic Diseases, College of Veterinary Medicine, South China Agricultural University, Guangzhou, China; ^3^Department of Biotechnology, Lovely Professional University, Phagwara, India; ^4^Zhaoqing Branch Center of Guangdong Laboratory for Lingnan Modern Agricultural Science and Technology, Zhaoqing, China; ^5^Department of Laboratory Medicine and Pathobiology, University of Toronto, Toronto, ON, Canada; ^6^Banting and Best Diabetes Centre, University of Toronto, Toronto, ON, Canada; ^7^Key Laboratory of Zoonosis Prevention and Control of Guangdong Province, Guangzhou, China

**Keywords:** influenza A viruses, H3N2 canine influenza virus (CIV), interspecies transmission, genetic change, positive selection, codon adaptation index

## Abstract

Since its first isolation in around 2007, the avian-origin H3N2 canine influenza virus (CIV) has become established and continues to circulate in dog populations. This virus serves as a useful model for deciphering the complex evolutionary process of interspecies transmission of influenza A virus (IAV) from one species to its subsequent circulation in another mammalian host. The present investigation is a comprehensive effort to identify and characterize genetic changes that accumulated in the avian-origin H3N2 CIV during its circulation in the dog. We revealed that H3N2 CIV experiences greater selection pressure with extremely high global non-synonymous to synonymous substitution ratios per codon (dN/dS ratio) for each gene compared to the avian reservoir viruses. A total of 54 amino acid substitutions were observed to have accumulated and become fixed in the H3N2 CIV population based on our comprehensive codon-based frequency diagram analysis. Of these substitutions, 11 sites also display high prevalence in H3N8 CIV, indicating that convergent evolution has occurred on different lineages of CIV. Notably, six substitutions, including HA-G146S, M1-V15I, NS1-E227K, PA-C241Y, PB2-K251R, and PB2-G590S, have been reported to play imperative roles in facilitating the transmission and spillover of IAVs across species barriers. Most of these substitutions were found to have become fixed in around 2015, which might have been a favorable factor that facilitating the spread of these CIV lineages from South Asia to North America and subsequent further circulation in these areas. We also detected 12 sites in six viral genes with evidence for positive selection by comparing the rates of non-synonymous and synonymous substitutions at each site. Besides, our study reports trends of enhanced ongoing adaptation of H3N2 CIV to their respective host cellular systems, based on the codon adaptation index analysis, which points toward increasing fitness for efficient viral replication. In addition, a reduction in the abundance of the CpG motif, as evident from an analysis of relative dinucleotide abundance, may contribute to the successful evasion of host immune recognition. The present study provides key insights into the adaptive changes that have accumulated in the avian-origin H3N2 viral genomes during its establishment and circulation into dog populations.

## Introduction

Influenza A virus (IAV) belongs to the family Orthomyxoviridae. According to the antigenicity of surface glycoproteins, namely, hemagglutinin (HA) and neuraminidase (NA), IAVs can be subtyped into multiple HxNy subtypes, including 18 HA (H1–H18) and 11 NA (N1–N11) (Mostafa et al., 2018). IAVs have been isolated from a wide range of animal hosts, including birds, humans, horses, whales, minks, pigs, and dogs, with specific subtypes predominating in each species, and these viruses constantly pose threats to both human and animal health (Yoon et al., 2014). Aquatic birds are thought to be the reservoir hosts for IAVs, except H17N10 and H18N11, which are bat-origin (Yoon et al., 2014).

The past two decades have witnessed the interspecific transmission and circulation of two IAV subtypes in dog populations [termed canine influenza virus (CIV)], namely, the equine-origin H3N8 influenza virus (EIV) and the avian-origin H3N2 influenza virus (AIV) (Borland et al., 2020). Emerging around 1999, the H3N8 strain variant of EIV was first isolated in the southeastern United States in 2004 and was the first IAV to cause epidemic disease in dogs (Crawford et al., 2005; Anderson et al., 2012). Since its emergence, H3N8 CIV circulated continuously among dogs in the United States until 2016 (Borland et al., 2020) and caused sporadic outbreaks in the dogs in the United Kingdom and Australia (Daly et al., 2008; Kirkland et al., 2010). Another CIV in canine, H3N2 CIV, was isolated from dogs around 2007 and rapidly spread into several areas of Southeast Asia (Li et al., 2010; Bunpapong et al., 2014). This CIV lineage then spread to the United States and Canada, through viral importation events from Asia in 2015 and 2017, respectively, subsequently causing more than 1,000 infections in dogs in these areas (Pulit-Penaloza et al., 2017; Voorhees et al., 2017, 2018; Weese et al., 2019). H3N2 CIV has now successfully colonized to become an enzootic virus throughout South-East Asia and North America and occasionally causes epizootics in pet and sheltered dogs (He et al., 2019; Supplementary Figure 1).

Evolution drives the cross-species transmission of IAVs (Guo et al., 2019). H3N2 CIV arose from a single cross-species transfer event, with all of its gene segments having an avian origin (Li et al., 2010; Zhu et al., 2015), and has successfully established and maintained its circulation in its new host environment. Thus, H3N2 CIV serves as an informative and relatively reliable model for understanding how IAVs emerge and adapt in new hosts. Many previous studies have focused on the phylogenetic history of H3N2 CIV (Zhu et al., 2015; Voorhees et al., 2017, 2018; He et al., 2019). In addition, several putative or confirmed adaptive mutations that separate H3N2 CIV from avian reservoir viruses or lead to antigenic changes among circulation clades had been reported previously (Yang et al., 2013; Lin et al., 2016; He et al., 2019; Lyu et al., 2019; Wu et al., 2021). Recently, the evolutionary dynamics on population size, selection pressure, and nucleotide substitution rates on virus genes of H3N2 CIV has been estimated (Shen et al., 2021). However, the genetic factors associated with its adaptation remain unclear. In the present study, we explored and thoroughly characterized the process by which H3N2 CIV adapted to its new host environment in real time (i.e., epidemiological time), after the spillover event from its avian source specie from multiple perspectives including analyses that assess selection pressure, amino acid mutation, codon usage, and dinucleotide distributions. Information provided by the present analysis, in combination with existing knowledge, promises to provide detailed insights into the potential genetic factors that may be responsible for host adaptation and facilitate an understanding of the complex patterns of adaptive evolution in establishing stable lineage descendants.

## Materials and Methods

### Dataset Collection and Processing

All influenza sequences used in this study were downloaded from the Influenza Virus Resource at the National Center for Biotechnology Information (NCBI)^1^ and the Global Initiative on Sharing Avian Influenza Data^2^. Redundant sequences, laboratory strains, and short sequences (<85% of the corresponding gene) were removed. MAFFT v7.221 was used to generate individual gene codon-based alignments followed by manual alignment to codon position. Specially, aligned nucleotide sequences of MP and NS segments were edited such that all of the codons from the first open reading frame (ORF) (M1 or NS1) were followed by the codons from the second ORF (M2 or NEP/NS2) to avoid repetition of nucleotides between these two ORFs. For the PA and PB1 segments, only the longest ORF was used.

#### Avian IAV Dataset

More than 18,000 avian-isolated complete genomes of IAVs were downloaded. For the HA and NA gene segments, only subtypes H3Nx and HxN2 were considered for further curation, respectively. The large datasets were reduced using methods applied in a previous study (Zhu et al., 2015) to make this analysis tractable. Specifically, gene-wise datasets were clustered based on an identity of greater than 98% using the CD-HIT software package (Li and Godzik, 2006), with one sequence from each cluster selected for the maximum likelihood (ML) phylogenetic analysis of all H3N2 CIV strains for each gene segment using IQ-TREE with default settings (Nguyen et al., 2015). Subsequently, the subset of avian sequences closely related to H3N2 CIV was selected and expanded to the original number of taxa (before using CD-HIT) (Zhu et al., 2015) for subsequent analyses. In total, 2867 taxa for PB2, 3410 taxa for PB1, 4407 taxa for PA, 3229 taxa for NS, 3311 taxa for NP, 2282 taxa for NA, 2564 taxa for MP, and 603 taxa for HA were used in the downstream analyses (Supplementary Table 1 and Supplementary Sheet 1).

#### H3N2 and H3N8 CIV Datasets

All available H3N2 CIV and H3N8 CIV strains were downloaded and pretreated as described above. Recombinant strains and some other lineages were excluded as previously described (Chen et al., 2018). The final dataset comprised of 264, 253, 261, 258, 252, 250, 247, and 247 unique HA, MP, NA, NP, NS, PA, PB1, and PB2 coding sequences, respectively, for H3N2 CIV (Supplementary Table 1 and Supplementary Sheet 2). For H3N8 CIV, the final dataset encompassed 108, 204, 93, 73, 203, 54, 55, and 56 unique HA, MP, NA, NP, NS, PA, PB1, and PB2 coding sequences, respectively (Supplementary Table 1 and Supplementary Sheet 3).

### Sequence Analysis

#### Selection Analysis

Rates of non-synonymous and synonymous substitutions (dN/dS) for each segment from H3N2 AIV, H3N2 CIV, and H3N8 CIV were estimated using the SLAC (Single Likelihood Ancestry Counting) method (Kosakovsky Pond and Frost, 2005) with ML trees inferred by IQ-TREE as the input reference trees. To test the potential impact of sampling bias on the results, a reshuffling test was performed. Specially, for each alignment, 50% of the sequences were randomly selected, and the SLAC analysis was performed based on the sub-datasets. The reshuffling test was performed three times for each alignment, and a similar trend among the reshuffled sub-datasets would suggest that sampling bias did not affect the results. SLAC, FEL (Fixed Impact Probability) (Kosakovsky Pond and Frost, 2005), MEME (Evolutionary Mixed Effects Model) (Murrell et al., 2012), and FUBAR (Fast, Unconstrained Bayesian Approximation) (Murrell et al., 2013) methods implemented at the DATAMONKEY^3^ server were used to identify codons under positive selection (Weaver et al., 2018). Sites that were reported by at least two methods were considered as positively selected. The significant result is chosen by *p*-value < 0.1 for SLAC, FEL, and MEME, and posterior probability > 0.9 for FUBAR (He et al., 2019).

#### Identification of Effective Substitutions Over the Epidemiological Time Period

The year-wise frequencies of amino acids at the codon level were computed for each gene segment of H3N2 CIV. For each gene segment, if the number of sequences for a year was less than 3, then they were manually combined with the sequences belonging to the next year in the dataset to reduce calculation bias. Subsequently, a “proportion switch” was defined as the replacement of one abundant amino acid by another in successive years, n(ω, j_th_, a_k_) was defined as the number of sequences with amino acid a_k_ at the j_th_ position, and the amino acid proportion *f* (ω, j_th_, a_k_) at the j_th_ position at year ω was given by *f* (ω, j_th_, a_k_) = n(ω, j_th_, a_k_)/N(ω) (Shih et al., 2007; Pu et al., 2018). A proportion switch between two different amino acids a_m_ and a_n_ at a given site j_th_ between years ω and ω + 1 was reported when both of the following conditions were met: (i) *f* (ω, j_th_, a_m_) + *f* (ω, j_th_, a_n_) > 0.8 and (ω + 1, j_th_, a_m_) + *f* (ω + 1, j_th_, a_n_) > 0.75 and (ii) [n(ω, j_th_, a_m_) – n(ω, j_th_, a_n_)] and [n(ω + 1, j_th_, a_m_) – n(ω + 1_th_, j, a_n_)] had opposite signs or zero in absolute value (Shih et al., 2007; Pu et al., 2018). Finally, we defined an “effective substitution” as a novel amino acid accumulating to a high proportion (>75%) in the following epidemiological time after the proportion switch.

#### Measurement of Codon Usage and Dinucleotide Distributions

ORFs were concatenated (HA+MP+NA+NP+NS +PA+PB1+ PB2) to assess the adaptation of the H3N2 CIV to the host microenvironment (Cristina et al., 2015). Base compositional features of the virus genome, including proportion of G and C at the first (GC1), second (GC2), and third (GC3) positions of codons and overall dinucleotide distribution, were calculated using the CodonW program^4^ (Supplementary Table 2). Dinucleotides with relative abundance > 1.25 were considered to be overrepresented, whereas dinucleotides with relative abundance < 0.78 were inferred as underrepresented (Kunec and Osterrieder, 2016). The GC3 values (*x*-axis) of the viral genes were then plotted against the respective GC12 values (mean of GC1 and GC2) (*y*-axis) to generate neutrality plots to explore the magnitude of the genomic compositional constraint and natural selection operating on virus genomes (Sueoka, 1988). To evaluate the fitness of the codon usage of H3N2 CIV to the host expression system, we employed the codon adaptation index (CAI) using the CAIcal web server^5^ (Sharp and Li, 1987; Puigbo et al., 2008), with host genomes (duck and canine) downloaded from the Ensembl database^6^, as references.

### Statistical Analysis

Statistical significance was evaluated using the Student’s *t*-test, with a significance level of 5% (0.05). To investigate the evolution of viral genome across the evolutionary timescale, linear regressions were performed between CAI values, dinucleotide abundance, and their respective collection dates. The presence of a significant regression coefficient was considered as supportive of adaptation over time (Franzo et al., 2017). All statistical analyses were performed using the SPSS software package (IBM Corp; version 23.0).

## Results and Discussion

**FIGURE 1:**
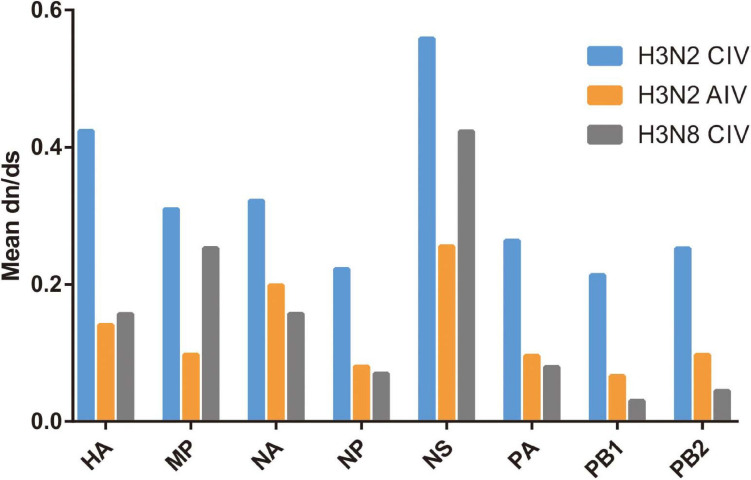
Comparative analysis of the global dN/dS ratio for each gene segment of H3N2 CIV (blue), H3N2 AIV (orange), and H3N8 CIV (gray) based on the completed alignments.

The subsequent spread of a newly emergent IAV within a new host population requires a period of adaptation (Guo et al., 2018). This study explores and characterizes the host evolution process of H3N2 CIV over its epidemiological time, after its interspecific transmission event from an avian IAV source and aims to unravel the riddles of the emergence and subsequent adaptation of an IAV in a new host environment. Here, supported by previous reports (Voorhees et al., 2018; He et al., 2019), our analysis revealed that with its establishment in canine populations, H3N2 CIV experiences greater levels of selection pressure than H3N2 AIV or H3N8 CIV, as evident from the significantly higher dN/dS substitution ratios seen for each gene (Figure 1). The reliability of our results was further demonstrated by the reshuffling test which revealed similar trends among the reshuffled sub-datasets (Supplementary Figure 2), indicating that sampling bias did not influence the results. Higher dN/dS ratios reflect both adaptive pressure and relaxed selective constraint that might facilitate the accumulation of favorable genetic changes associated with adaptive finesse to a new host population (Joseph et al., 2018). Similarly, changes in adaptive pressure leading to higher dN/dS rations have also been reported for both H2N2 pandemic strains and European avian-like H1N1 swine influenza virus (SIV) strains during their evolution after separating from their reservoir avian IAVs strains (Joseph et al., 2015, 2018).

**FIGURE 2:**
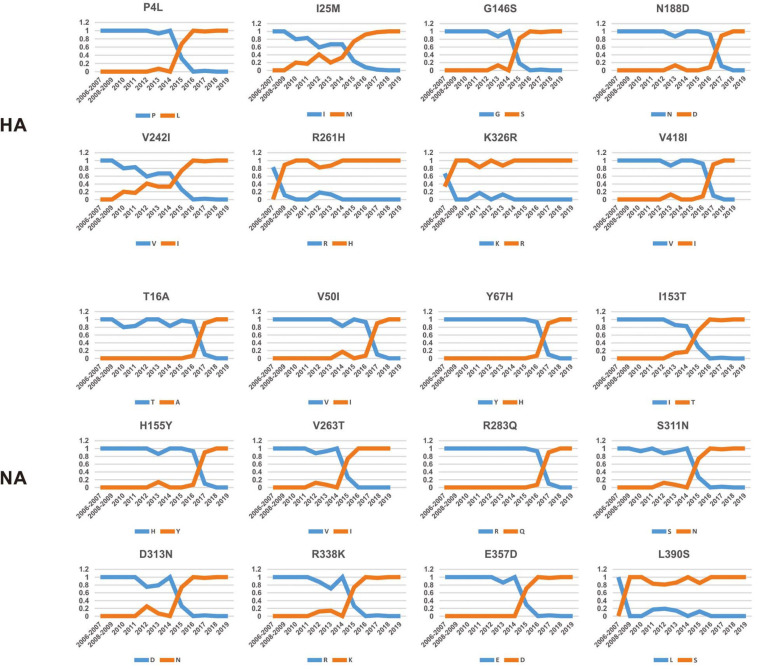
Dynamic changes in the amino acid frequencies for sites in each effective substitution site in the HA and NA gene segments of H3N2 CIV during the circulation phase.

To investigate how the adaptive pressure acts on the evolution of H3N2 CIV, we undertook a comprehensive frequency diagram analysis by real-time scanning of amino acid frequency changes at the codon level for each gene. Such investigations tend to be useful in visualizing the temporal dynamics of substitutions reflecting evolution at any amino acid site (Shih et al., 2007). A total of 54 effective substitutions were observed during the circulation of H3N2 CIV (Table 1, Figure 2, and Supplementary Figure 3), signifying the potential functional importance of these sites and the impact of positive selection on them. Notably, most of these effective substitutions displayed extremely low frequency in H3N2 AIV (Table 1), which implies an increasing genetic distance between H3N2 CIV and its reservoir viruses. Some of the effective substitutions, such as HA-V418I, NA-L390S, M2-R18K, NS1-E227K, and PB2-K251R, were also seen in the majority of the AIV sequences with a proportion of greater than 75% (Table 1). We speculate that mutations had occurred during the host-shift event of H3N2 CIV, but later reversed back, possibly due to selection pressures operational at different stages of the circulation phase of this lineage of newly acquired CIV (Long et al., 2019). However, it is interesting to note that 11 out of the 54 (20.37%) effective substitutions in H3N2 CIV, including HA-G146S, HA-V242I, HA-V418I, M1-V15I, M2-R18K, PA-N347D, PB1-V200I, PB2-K251R, PB2-I292T, PB2-V511I, and PB2-G590S, are fixed in H3N8 CIV (Table 1), indicating that convergent evolution has occurred on different lineages of CIVs at these sites. Such convergent sites may act as host markers associated with adaptation of IAVs in canine population and demand sincere attention.

**TABLE 1:** Effective substitutions that occurred during the circulation of H3N2 CIV.

Gene	Codon position^a^	H3N2 AIV proportion	H3N8 CIV proportion	Experimental validation^b^	Location/phenotype^c^
HA	P4L	1.82% (11/603)	0.00% (0/108)		
	I25M	1.16% (7/603)	0.00% (0/108)		
	**G146S**	0.00% (0/603)	100.00% (108/108)	S146G	Antigenic epitope A Potential to alter the virulence of H1N1pdm09 in swine (Henningson et al., 2015)
	N188D	11.44% (69/603)	0% (0/108)	S188N	Antigenic epitope B, Receptor-binding site (190 helix) Increased virulence in mammals (Matos-Patron et al., 2015)
	**V242I**	5.64% (34/603)	98.15% (106/108)		Antigenic epitope D
	R261H	0.83% (5/603)	0.00% (0/108)		
	K326R	0.83% (5/603)	0.00% (0/108)		
	**V418I**	84.25% (508/603)	100.00% (108/108)		
NA	T16A	1.67% (38/2282)	N/A		Transmembrane helix
	V50I	1.10% (25/2282)			
	Y67H	29.10% (664/2282)			
	I153T	0.00% (0/2282)			Located near catalytic sites 151 and 152 (McAuley et al., 2019).
	H155Y	20.60% (470/2282)			
	V263T	0.04% (1/2282)			
	R283Q	7.80% (178/2282)			
	S311N	7.93% (181/2282)			
	D313N	0.35% (8/2282)			
	R338K	35.89% (819/2282)			
	E357D	0.00% (0/2282)			
	L390S	96.23% (2196/2282)			
M1	**V15I**	1.56% (40/2564)	90.20% (184/204)	V15I/T	Increased virulence in mammals (Chen et al., 2007)
	R95K	0.59% (15/2564)	4.90% (10/204)		
	S207N	0.90% (23/2564)	4.90% (10/204)		
M2	G14E	2.11% (54/2564)	0.00% (0/204)		
	**R18K**	94.27% (2417/2564)	100.00% (204/204)		
	V27I	5.81% (149/2564)	4.90% (10/204)		
NS1	T197I	1.30% (42/3229)	0.49% (1/203)		
	P212S	1.95% (63/3229)	2.96% (6/203)		
	E227K	85.60% (2764/3229)	0.00% (0/203)	E227R	Human host marker (Finkelstein et al., 2007)
NS2	D27G	5.33% (172/3229)	0.00% (0/203)		
	L40F	1.18% (38/3229)	0.49% (1/203)		
PA	Y65H	0.00% (0/4407)	0.00% (0/54)		
	C241Y	0.32% (14/4407)	0.00% (0/54)	C241Y	Enhance the replicative ability of an H5N1 virus in A549 cells and enhance its pathogenicity in mice (Yamaji et al., 2015)
	E243D	0.02% (1/4407)	0.00% (0/54)		
	**N347D**	0.25% (11/4407)	96.30% (52/54)		
	S388G	58.13% (2562/4407)	0.00% (0/54)		
	R401K	0.59% (26/4407)	0.00% (0/54)		
	G684E	0.43% (19/4407)	0.00% (0/54)		
PB1	E97K	0.62% (21/3410)	0.00% (0/55)		
	R187K	0.09% (3/3410)	0.00% (0/55)	R187K	May contribute higher pathogenicity in mice for H9N2 (Liu et al., 2016)
	**V200I**	7.39% (252/3410)	94.55% (52/55)		
	S216N	4.13% (141/3410)	0.00% (0/55)		
	V218I	0.06% (2/3410)	0.00% (0/55)		
	T434S	0.00% (0/3410)	0.00% (0/55)		
	A661T	0.18% (6/3410)	0.00% (0/55)		
PB2	M76I	0.14% (4/2867)	0.00% (0/56)		
	**K251R**	97.84% (2805/2867)	92.86% (52/56)	K251R	Increased virulence in mice (Prokopyeva et al., 2016)
	**I292T**	11.58% (332/2867)	94.64% (53/56)		
	S334N	0.17% (5/2867)	0.00% (0/56)		
	V338I	7.12% (204/2867)	0.00% (0/56)		
	**V511I**	9.66% (277/2867)	100.00% (56/56)		
	**G590S**	2.55% (73/2867)	96.43% (54/56)	GQ590/591SR/K	Increased polymerase activity (Mehle and Doudna, 2009)
	T598A	0.70% (20/2867)	0.00% (0/56)		
	S714I	0.00% (1/2867)	0.00% (0/56)	S714R	Increased polymerase activity, increased virulence in mammals, mammalian host marker (Gabriel et al., 2005; Gabriel et al., 2007)

*^*a*^Mutations observed to be fixed during the circulation of H3N2 CIV. Mutations showing higher proportion in H3N8 CIV (>75%) are marked with bold. ^*b*^Experimental validation of mutations provided in previous studies. ^*c*^Associated location or phenotype of the mutations validated by previous studies.*

Intriguingly, 41 of the 54 effective substitutions (75.92%) took less than 5 years since their first occurrence to become fixed (Supplementary Figure 4), which is considerably less than the conditional fixation time for a neutral mutation (which is expected to 219 years) (Li, 1997). Such observations emphasize the important role of positive selection operating on these residues (Li, 1997). In addition, it is noteworthy that most of these substitutions were fixed around 2015 (Figure 2 and Supplementary Figure 3), a time when H3N2 CIV was spreading from South-East Asia to North America (Pulit-Penaloza et al., 2017; Voorhees et al., 2017, 2018; Weese et al., 2019). Whether the accumulation of these substitutions has contributed to the further expansion of this lineage of CIV needs to be further investigated. Besides, considering the high genetic similarity between the H3N2 CIV sequences recently isolated in South-East Asia and North America (Lyu et al., 2019), it is possible that a novel favorable genotype consisting of these adaptive mutations might have become dominant in both South-East Asia and North America. Our conjecture is also supported by the observation that most of these substitutions have become fixed with proportions extremely close to 1 (Figure 2 and Supplementary Figure 3) in recent years, indicating that there is little regional difference in these substitutions.

**FIGURE 3:**
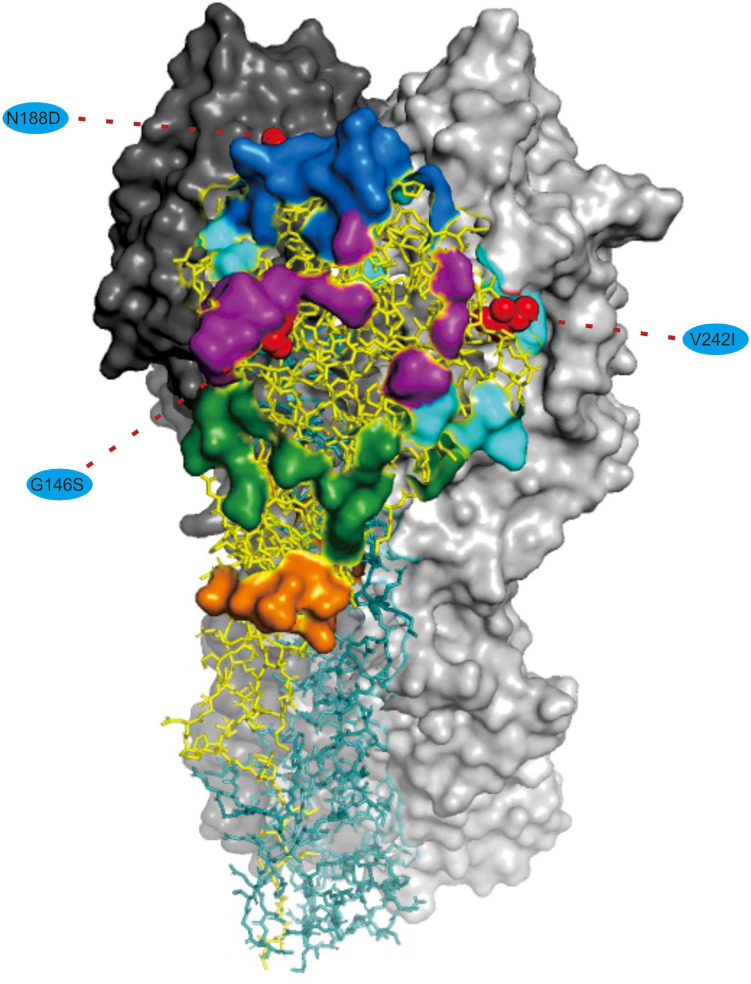
Mapping of selected effective substitutions onto the known 3D structure of HA (PDB ID: 6N4F). The monomers representing the HA1 and HA2 subunits are represented in yellow and blue, respectively. A trimer complex is shown in surface representation with the antigenic sites highlighted. Antigenic epitopes A, B, C, D, and E are marked in purple, blue, orange, sky blue, and green, respectively. Numbers in colored ellipse represent codon alignment number (H3 numbering). The G146S, N188D and V242I substitutions are shown as red spheres that are located at the antigenic epitopes A, B, and D, respectively.

We noted that 20 of the 54 (37.03%) effective substitutions occurred in the HA and NA genes (Table 1, Figure 2, and Supplementary Figure 3), which suggests that a greater level, relative to other viral genes, of adaptive pressure has acted on these two genes. This suggestion is also supported by our dN/dS analysis (Figure 1). HA is responsible for the attachment of the virus to the sialic acid receptors on the cell surface, whereas NA catalyzes the separation of the new virion from infected cells and facilitates viral movement through mucus (Mostafa et al., 2018). It has been reported that a functional optimal balance between HA and NA is required for efficient replication and transmission of IAVs (Mitnaul et al., 2000; Yen et al., 2011). The N188D substitution, located at the receptor-binding region (190 helix) of HA protein (Figure 3), is a notable replacement (Skehel and Wiley, 2000). A recent study revealed that alterations of amino acid at this site have significant effects on the binding capacity of A(H1N1)pdm09 (Matos-Patron et al., 2015). In addition, N188D, G146S, and V242I mutations are located at the antigenic epitope B, antigenic epitope A, and antigenic epitope D of the HA protein, respectively (Figure 3), where substitutions at these sites may lead to antigenic drift in IAVs and the subsequent evasion of host immune responses (Shih et al., 2007).

**FIGURE 4:**
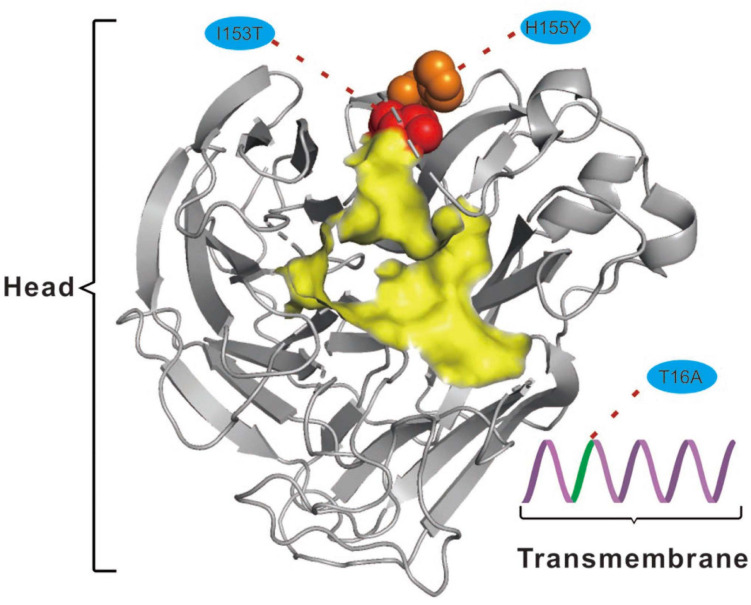
Mapping of selected effective substitutions onto the known 3D structure of NA (PDB ID: 6N4D). The I153T (represented as a red sphere) and H155Y (represented as an orange sphere) substitutions are located close to the active site (highlighted in yellow) on the head region (shown as gray cartoon) of the NA protein. The T16A (shown as green cartoon) substitution is located in the transmembrane region (shown as a purple cartoon) of the NA protein.

For the NA protein, a T16A substitution occurs at a site located in the transmembrane region of this protein (Figure 4; McAuley et al., 2019). The transmembrane region anchors the NA protein to membrane and is considered to be part of the signal peptide that permits the transport of this protein across the endoplasmic reticulum membrane (McAuley et al., 2019). Therefore, adaptive mutations in this region might alter the interactions of the NA protein to the membrane for better stability in new hosts. Interestingly, this site had previously been reported to be under positive selection in avian-origin European H1N1 SIV strains (Joseph et al., 2018), thus signifying a potential functional role of the amino acid residues at this site in the adaptation of IAVs between avian and mammalian hosts. In addition, the I153T and H155Y are located close to the enzymatic site of the NA protein (Figure 4; McAuley et al., 2019). Variation in this enzymatic region, and relative framework residues, tends to change the aspects of viral replication, transmissibility, and its susceptibility to antiviral inhibitors (McAuley et al., 2019). Taken together, mutations at these functionally important sites in HA and NA might play crucial roles in regulating receptor-binding specificity and affinity, as well as facilitating evasion from host antibodies, thus conferring enhanced fitness to H3N2 CIV in new host environments. However, functional experiments are needed to reach definite conclusions.

Effective evasion from host immune responses along with rapid viral replication and assembly are other factors critical to the virulence and host range of IAVs (Long et al., 2019). Replication and transcription of influenza viruses are catalyzed by the viral polymerase complex composed of the PB2, PB1, and PA proteins, while the M1 protein plays an essential role in viral assembly, budding, and morphogenesis, and the NS1 protein is the major viral interferon antagonist for IAVs (Mostafa et al., 2018). In the present study, we observed that multiple adaptive mutations have occurred and have been fixed in these genes that might play crucial roles in facilitating the transmission and spillover of IAVs across species barriers (Table 1). The NS1-227K/R had been reported to be a mammalian marker and exists in all human pandemic influenza viruses while NS1-227E has been noted to be highly conserved in avian strains (Finkelstein et al., 2007). Given the role of the NS1 protein in suppressing host cellular immune responses, the NS1-E227K mutation might be a molecular determinant to hone the function of the immune suppression by H3N2 CIV. In addition, M1-V15I (Chen et al., 2007), PA-C241Y (Yamaji et al., 2015), PB1-R187K (Liu et al., 2016), PB2-K251R (Prokopyeva et al., 2016), and PB2-G590S (Mehle and Doudna, 2009) substitutions have all been reported to enhance pathogenicity and virulence of AIVs in mammalian hosts due to their contributions to improving replicative efficacy and assembly fitness. Notably, no substitution was found to be fixed in the NP gene, which may indicate that the adaptation of H3N2 CIV to canines is less reflected by changes in the NP segment (Table 1). Such an observation is consistent with higher selective constraints (as evident from the lower dN/dS ratio) in the NP gene compared with other gene segments (Figure 1).

Analyses to identify sites being suffered by positive selection in viruses have been wildly used to characterize signatures of adaptive evolution (Joseph et al., 2015, 2018; He et al., 2019). He et al. (2019) identified four positively selected sites (4, 218, 436, and 453) in HA and one positively selected site (222) in NA for H3N2 CIV using the dN/dS method. The present analysis involves a larger sequence dataset associated with H3N2 CIV whole genomes and identified 12 residues in six viral gene segments that are suggested to have experienced positive selection (Table 2). Of these 12 sites, three (HA-436, HA-453, and NA-222) were previously reported by He et al. (2019). Interestingly, real-time scanning of amino acid frequency at these sites, both from the current study and from the report by He et al. (2019), clearly demonstrates that no significant change in frequency has occurred at these sites, with the exception of HA-4 (Supplementary Figure 5). Although sites M2-22, M2-29, NA-36, and NA-222 had undergone one or more substitutions, none have been fixed in the examined circulation time, in conflict with the expectations of positive selection acting on these residues. On the other hand, most of the effective substitutions observed in the current study were not detected as having signals of positive selection with the dN/dS method. These observations highlight the sensitivity and robustness of the frequency diagram analysis method in revealing footprints of natural selection in comparison to inferences derived from dN/dS analysis.

**TABLE 2:** Sites under positive selection in the H3N2 CIV gene segments.

		Test methods							
		MEME		FAUBAR		FEL		SLAC	
Segment	Codon	ω^+^	*p*-value	dN-dS	Post.Pro	dN-dS	*p*-value	dN-dS	*p*-value
HA	141	NS		3.602	0.906	3.004	0.083	ns	
	436	3.66	0.08	5.632	0.943	3.353	0.067	ns	
	453	5.38	0.03	8.087	0.987	5.244	0.022	10	0.0609
M1	15	3.36	0.09	8.87	0.984	3.36	0.067	ns	
M2	22	6.58	0.02	11.762	0.987	6.582	0.01	12.6	0.0642
	29	3.51	0.08	5.444	0.959	ns		ns	
NA	36	3.21	0.1	4.943	0.96	3.208	0.073	ns	
	222	NS		3.665	0.901	2.842	0.092	ns	
NS1	71	3.25	0.09	6.967	0.963	3.251	0.071	ns	
PA	99	NS		4.911	0.953	3.076	0.079	ns	
	237	NS		4.787	0.935	2.762	0.097	ns	
PB1	723	4.06	0.06	4.67	0.953	4.059	0.044	ns	

*Position in HA is based on the H3 numbering. MEME *p* < 0.1, FEL *p* < 0.1, SLAC *p* < 0.1, FAUBAR Post.Pro > 0.9. NS, non-significant.*

**FIGURE 5:**
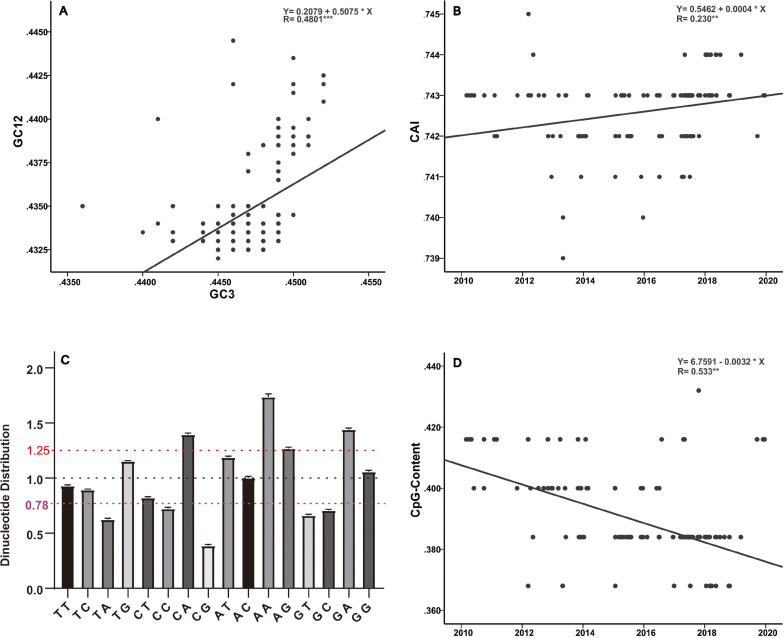
Base composition analysis of the H3N2 CIV genomes. **(A)** GC3 values are plotted against the GC12 values to generate a neutrality plot. **(B)** CAI values calculated with respect to the canine host are plotted according to collection date. **(C)** Relative dinucleotide abundance analysis for the H3N2 CIV. Dotted lines in red, black, and purple signify the optimal values for overrepresentation, average representation, and underrepresentation of the dinucleotides, respectively. **(D)** CpG contents plotted according to sampling date of the H3N2 CIV.

Host selection pressure on viral replicative efficacy and adaptation leaves a footprint in the base composition of a viral genome (Greenbaum et al., 2008; Guo et al., 2020). The genetic code is degenerate, and the non-random usage of synonymous codons leads to codon usage bias (Shah and Gilchrist, 2011). Codon usage is believed to be mainly shaped by a “mutation-selection-drift balance” (Shah and Gilchrist, 2011). Viruses, owing to their small genomes, rely on host cellular machinery for processes such as replication and protein synthesis and show a strong relationship with host codon usage. It is believed that a higher similarity of their codon usage with the host codon usage profile provides them with an advantage as they would be adapted to host tRNA pool and thus could efficiently generate new progeny (Tian et al., 2018). Such conjectures have been verified by previous functional experiments (Carlini, 2004; Mueller et al., 2006; Coleman et al., 2008). In addition, it has been reported that current H3N2 viruses enhance replication in interferon (IFN)-treated human cells, by gradually skewing codon usage toward the interferon-altered tRNA pool (Smith et al., 2018). In the present analysis, the slope of the regression line pertaining to the neutrality plot between GC3 values (*x*-axis) and GC12 (*x*-axis) of the H3N2 CIV genes is 0.5075 (Figure 5A). It has been suggested that the slope of these plots reflects the degree of compositional constraint operating on the genes of interest (Sueoka, 1988). Such an observation indicates that both of the compositional constraint (50.75%) and natural selection (49.25%) have a similar magnitude in influencing the codon usage of H3N2 CIV genomes. CAI analysis reveals the viral adaptability in codon usage to that of corresponding host(s), and a higher CAI indicates strong adaptation to host cellular machinery (Sharp and Li, 1987). Consistent with a previous study (Li et al., 2018), we found that H3N2 CIV exhibited better adaptation to the duck’s codon usage pattern than that of the canine, which is evident from the observation that H3N2 CIV showed a significantly higher mean CAI value to the duck host than that to the canine host (0.818464 ± 0.000953 for duck host versus 0.742636 ± 0.000894 for canine host, *p* < 0.001) (Supplementary Table 2), possibly due to a long-term circulation in the avian environment before transmission to canines. However, the present findings reveal a reduction in the distance between H3N2 CIV and the canine codon bias over time (Figure 5B), thus indicating increasing fitness to the canine usage pattern for H3N2 CIV. Similar observations have been reported for canine parvovirus type 2 and H1N1/pdm2009 viruses, where the host shift event from their reservoir host to the new host environment leads to subsequent adaptation in codon usage to better fit the cellular machinery of the new hosts (Franzo et al., 2017; Guo et al., 2020).

Analysis of the relative abundance of the 16 possible dinucleotides in the H3N2 CIV genomes revealed a distinct usage preference among the dinucleotides (Figure 5C and Supplementary Table 2). It was noted that the CpA, ApA, and GpA dinucleotides were significantly overrepresented (relative abundance > 1.25). In contrast, TpA, CpC, GpT, GpC, and CpG dinucleotides were significantly underrepresented (relative abundance < 0.78). Notably, only the CpG dinucleotide was observed to be extremely underrepresented (relative abundance < 0.50) (Figure 5C and Supplementary Table 2). Extensive analyses of the relative abundance of the CpG dinucleotide over epidemiological time showed that H3N2 CIV has experienced a continuous decrease in CpG dinucleotide content (Figure 5D). It is believed that CpG motifs in viral genomes are treated as viral molecular patterns and are recognized by the host’s intracellular pattern recognition receptor Toll-like receptor 9 (TLR9), leading to the stimulation of immune responses against the viral pathogens. Thus, suppression of CpG dinucleotides points toward a strategy by the viruses to avoid host immune signals (Sivori et al., 2004; Jimenez-Baranda et al., 2011). In addition, a tendency of sustained reduction of CpG motifs in the H3N2 CIV genome is indicative of these viral pathogens continuing to improve their response to evade host immune signals (Figure 5D and Supplementary Table 2). Such observations have previously been reported for the 1918 human H1N1, 1968 human H3N2, and 1963 horse H3N8 viruses during their circulation phases (Greenbaum et al., 2008; Jimenez-Baranda et al., 2011).

## Conclusion

Influenza A virus endemics in animals such as canines and cats should be carefully monitored due to their large global population, with many of them having close contact with humans, thus they pose potential risks to public health. Recently, in 2016, a veterinarian in a New York animal shelter was infected through a transmission event for a feline H7N2 influenza virus (Belser et al., 2017). Such instance forewarns potential viral transmission through close contact between humans and their companion animals (Belser et al., 2017). In addition, the fact that the respiratory tract of dogs harbors receptors for both avian-adapted and mammal-adapted influenza viruses (Tate et al., 2014) and the increasing evidence of canine infection with swine, human, and avian IAVs of different subtypes (Chen et al., 2018; Zhao et al., 2020) point toward a capacity for canines to serve as “mixing vessels” to generate novel gene constellations, which may lead to future pandemics in the human population.

The present study deals with the identification and profiling of genetic changes during the process of adaptation of H3N2 CIV over epidemiological time. We observed that H3N2 experiences a higher level of adaptive selection pressure that yielded 54 effective substitutions that became fixed in the viral population. Some of these mutations have been experimentally verified to play important roles in allowing AIVs to cross species barriers, while others should be treated as candidate sites for further functional experiments. Most of the substitutions became fixed around 2015, which might have facilitated the invasion and further circulation of this lineage of CIV from South Asia to North America. In addition, the present findings revealed evidence of convergent evolution in the different CIV lineages. We also detected multiple sites under positive selection using the dN/dS analysis method and showed that the frequency diagram analysis method might exhibit higher sensitivity and robustness for revealing the impact of natural selection. Codon usage analysis, as executed in the present study, indicated that H3N2 CIV is better adapted to the duck’s cellular machinery, in comparison to canine host, thus supporting existing knowledge regarding the transmission route of the virus from an avian to canine host. In the present study, an increasing fitness of the virus to the host microenvironment, following its emergence in the canine population, was also detected by employing codon usage and dinucleotide distribution analyses. Such observations reveal that the adaptive evolution of H3N2 CIV, after its original incursion, further increased adaptation to the canine host environment.

## Data Availability Statement

The original contributions presented in the study are included in the article/Supplementary Material, further inquiries can be directed to the corresponding author/s.

## Author Contributions

YS designed and supervised the study. FG, AR, RW, JY, ZZ, WL, and XS generated the data. FG analyzed the data. FG, YS, AR, and DI wrote and prepared the manuscript. R-AC commented on and revised the drafts of the manuscript. All the authors have read and agreed to submission of the manuscript.

## Conflict of Interest

The authors declare that the research was conducted in the absence of any commercial or financial relationships that could be construed as a potential conflict of interest.

## Footnotes

1 www.ncbi.nlm.nih.gov/genomes/FLU

2 www.gisaid.org

3 http://www.datamonkey.org/analyses

4 http://sourceforge.net/projects/codonw/

5 http://genomes.urv.es/CAIcal/

6 http://www.ensembl.org

## Supplementary Material

Supplementary Figure 1 Regional distribution of the 301 H3N2 CIV strains isolated from 2005 to 2019 analyzed in the present study.

Supplementary Figure 2 Comparative analysis of the global dN/dS ratio for each gene segment of H3N2 CIV (blue), H3N2 AIV (orange), and H3N8 CIV (gray) based on the reshuffled sub-datasets represented by circles.

Supplementary Figure 3 Dynamic changes in the amino acid frequency for each effective substitution of the MP, NS, PA, PB1, and PB2 gene segments of H3N2 CIV during the circulation phase.

Supplementary Figure 4 Time required (years) for the fixation of 54 effective substitutions in H3N2 CIV since their first occurrence (only continuous accumulations have been considered).

Supplementary Figure 5 Dynamic changes in amino acid frequency for sites under positive selection (dN/dS > 1) during the circulation phase of H3N2 CIV. ^a^Positive selection sites detected in current study. ^b^Positive selection sites detected by He et al. (2019).

Supplementary Table 1 Strain information (strain name, accession number, host, subtype, location) for the selection and amino acid frequency analyses associated with AIV (Sheet 1), H3N2 CIV (Sheet 2), and H3N8 CIV (Sheet 3). Actual sequence number for each dataset that were used for identifying effective substitutions over epidemiological time for H3N2 CIV has also been provided in Sheet 4.

Supplementary Table 2 Strain name, collection date, digital date, location, base composition and CAI values for 220 completely sequenced H3N2 CIV genomes.
