# Supplementary figures and images for: Host Adaptive Evolution of Avian-Origin H3N2 Canine Influenza Virus
Source: Front Microbiol. 2021 Jun 14;12:655228. doi: 10.3389/fmicb.2021.655228 (PMC8236823; doi:10.3389/fmicb.2021.655228)

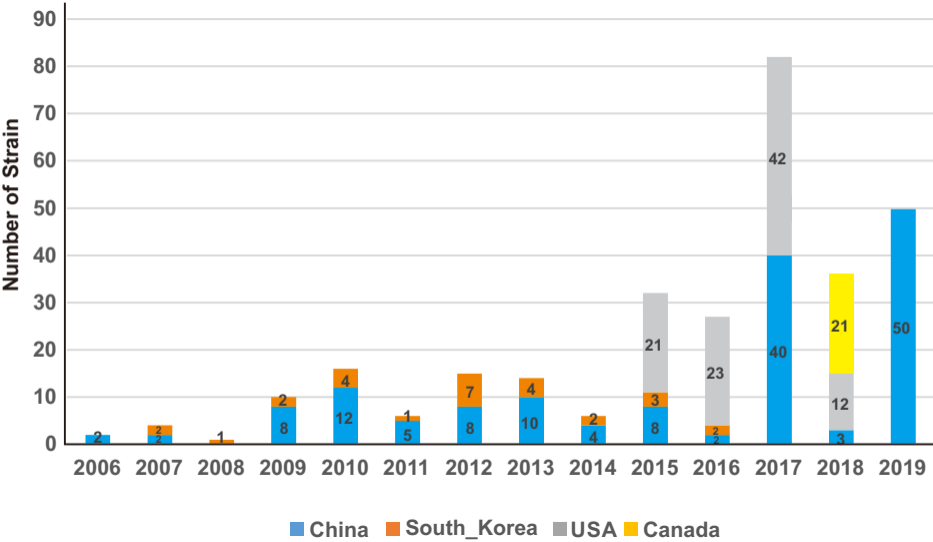

Supplement: Supplementary Figure 1 — Regional distribution of the 301 H3N2 CIV strains isolated from 2005 to 2019 analyzed in the present study. [file Data_Sheet_1.PDF]

Reshuffling test 1

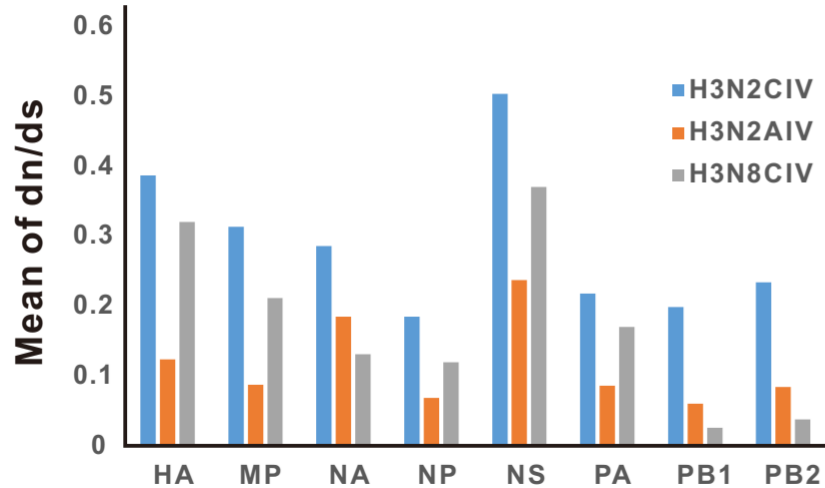

Reshuffling test 2

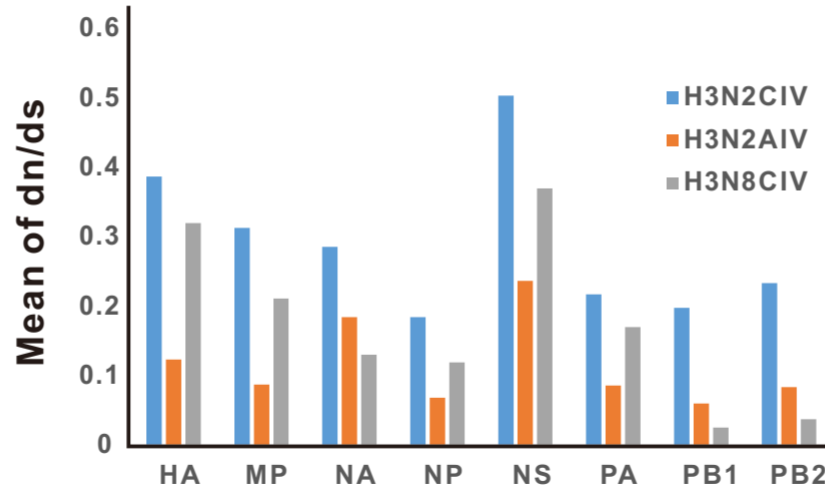

Reshuffling test 3

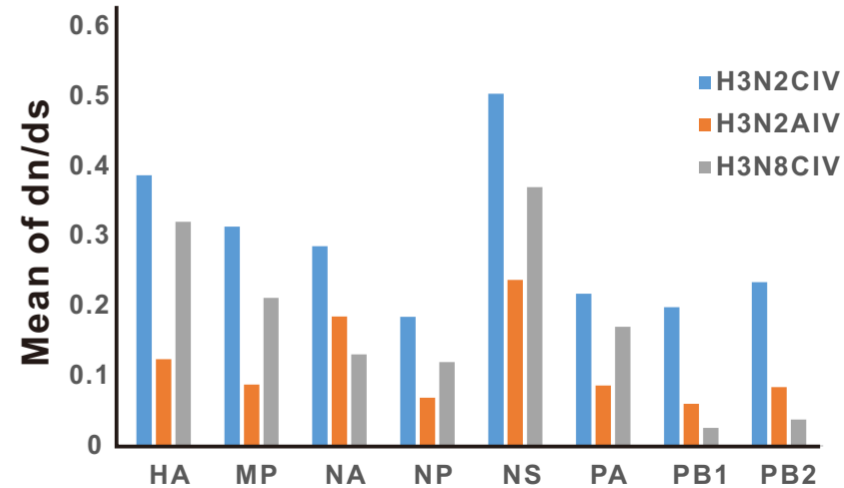

Supplement: Supplementary Figure 2 — Comparative analysis of the global dN/dS ratio for each gene segment of H3N2 CIV (blue), H3N2 AIV (orange), and H3N8 CIV (gray) based on the reshuffled sub-datasets represented by circles. [file Data_Sheet_2.PDF]

PA

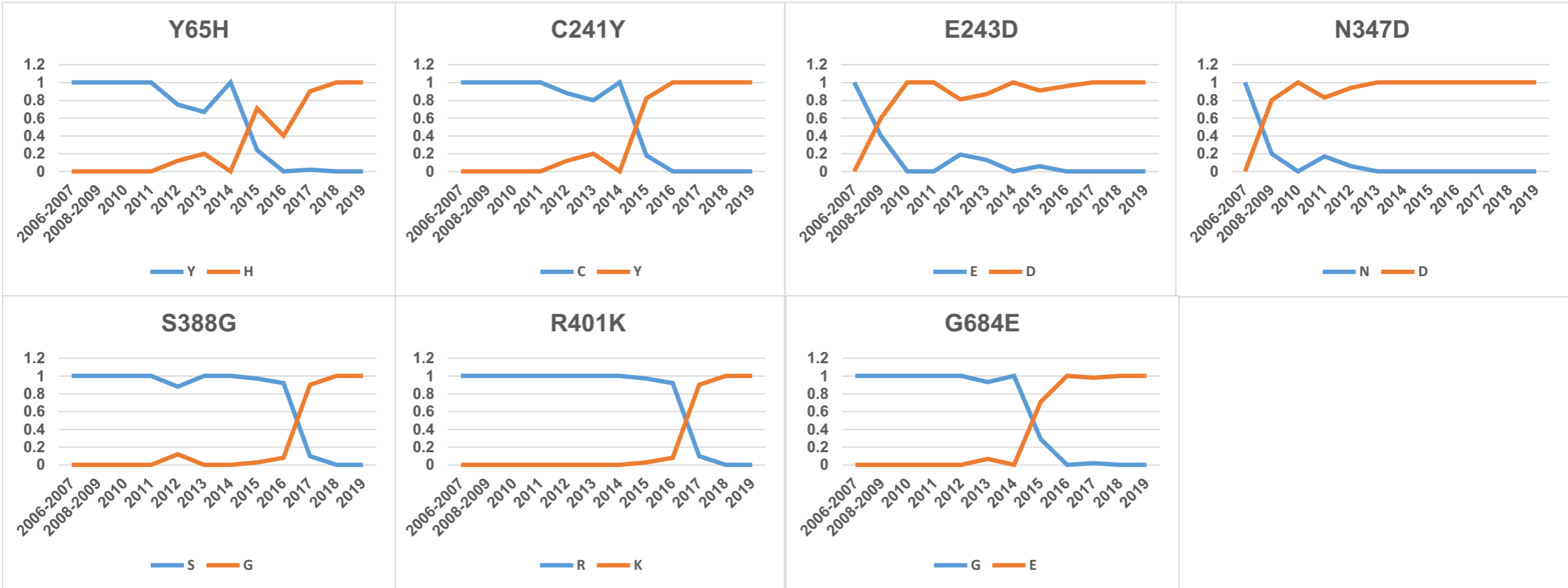

PB1

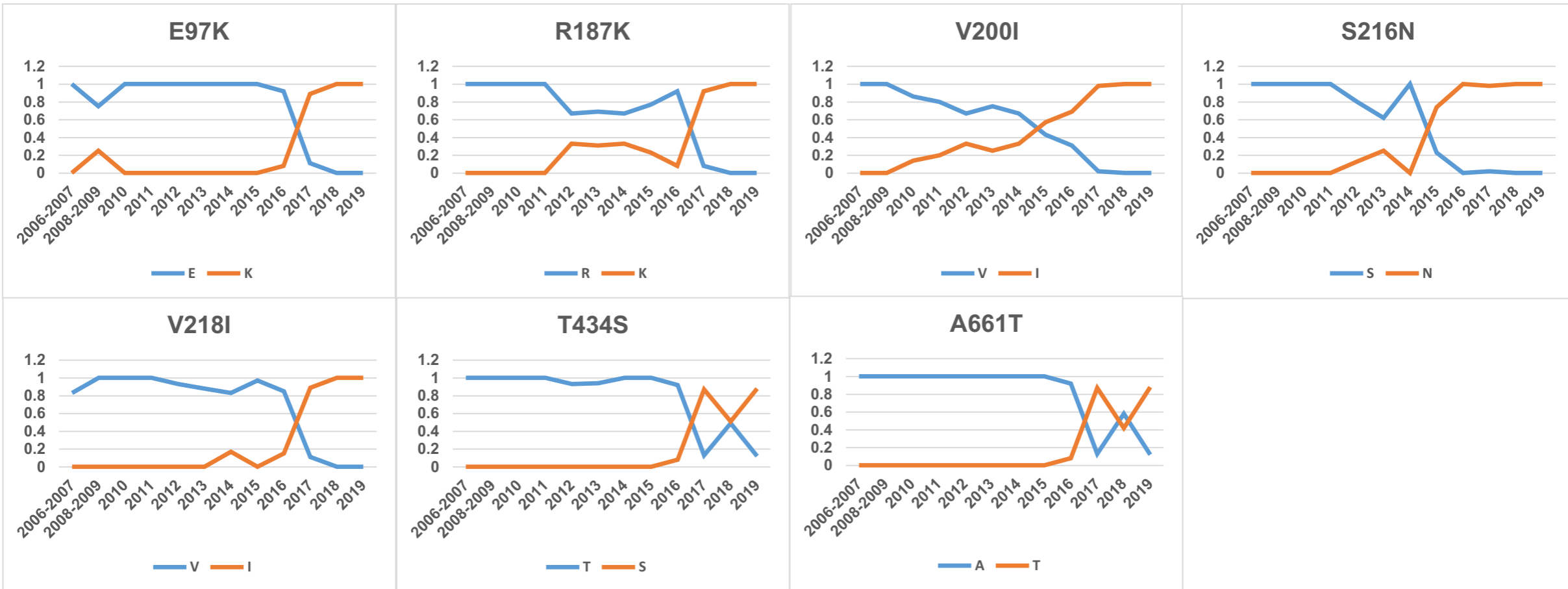

PB2

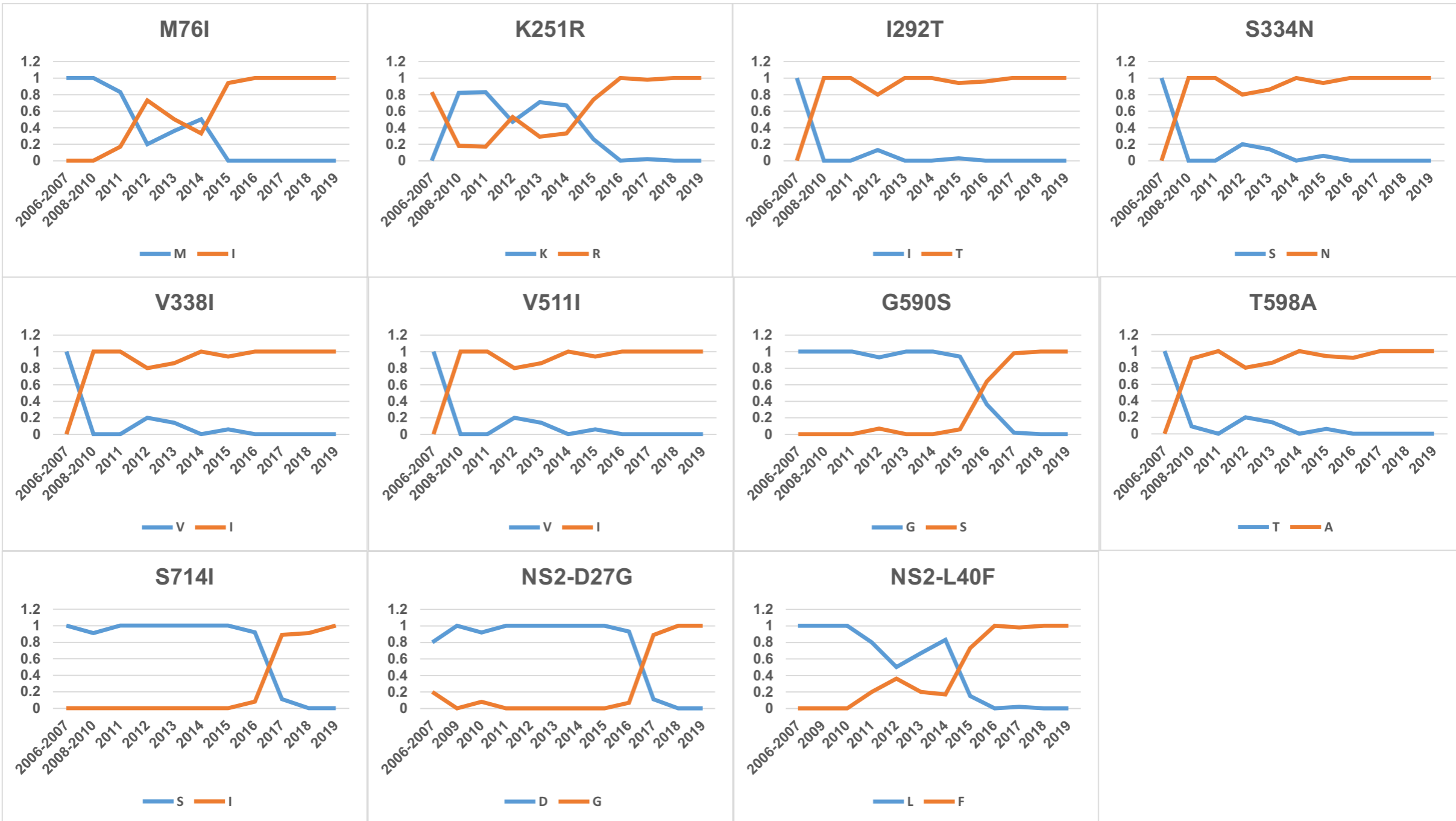

M1

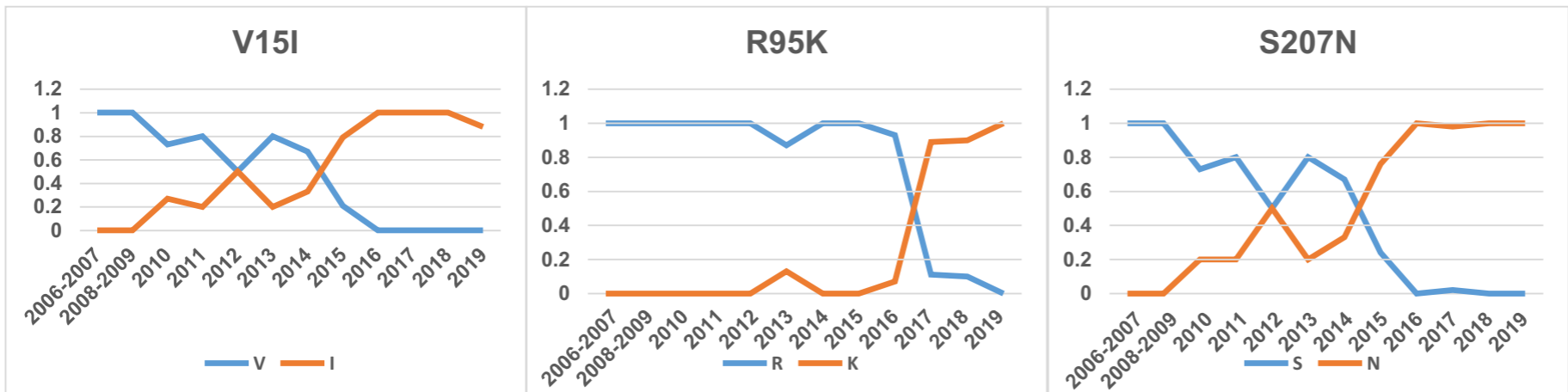

M2

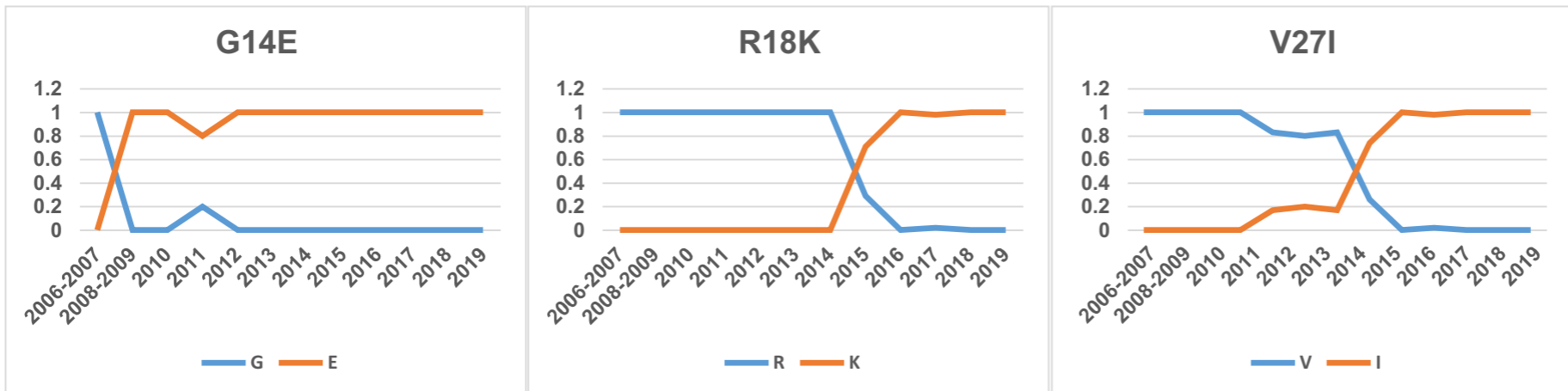

NS1

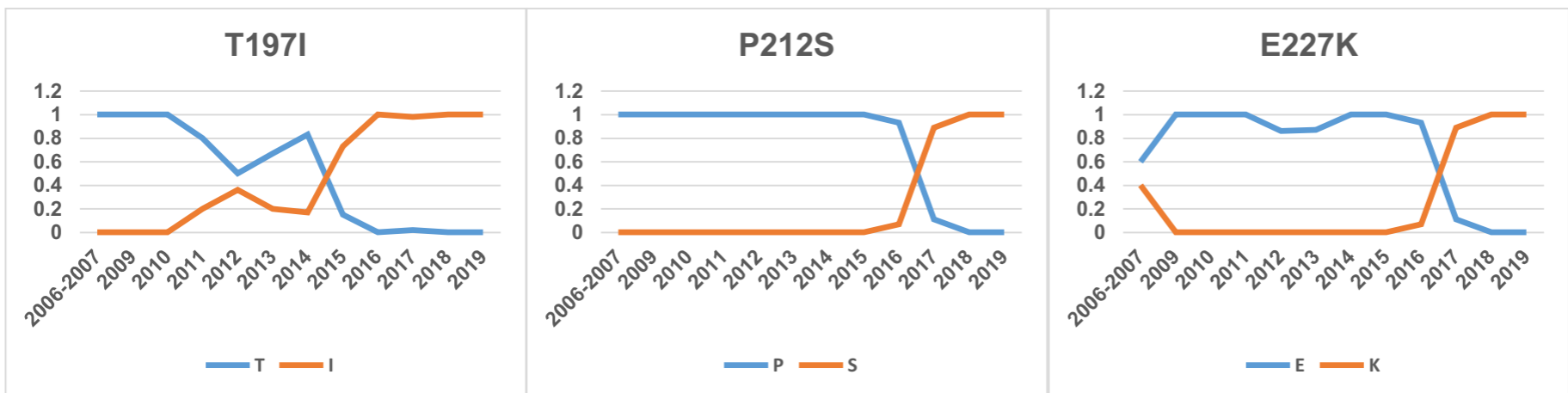

Supplement: Supplementary Figure 3 — Dynamic changes in the amino acid frequency for each effective substitution of the MP, NS, PA, PB1, and PB2 gene segments of H3N2 CIV during the circulation phase. [file Data_Sheet_3.PDF]

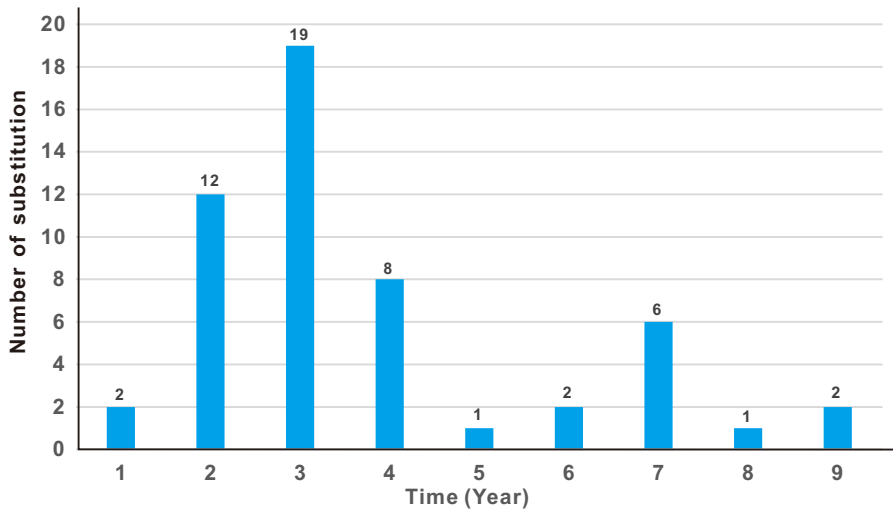

Supplement: Supplementary Figure 4 — Time required (years) for the fixation of 54 effective substitutions in H3N2 CIV since their first occurrence (only continuous accumulations have been considered). [file Data_Sheet_4.PDF]

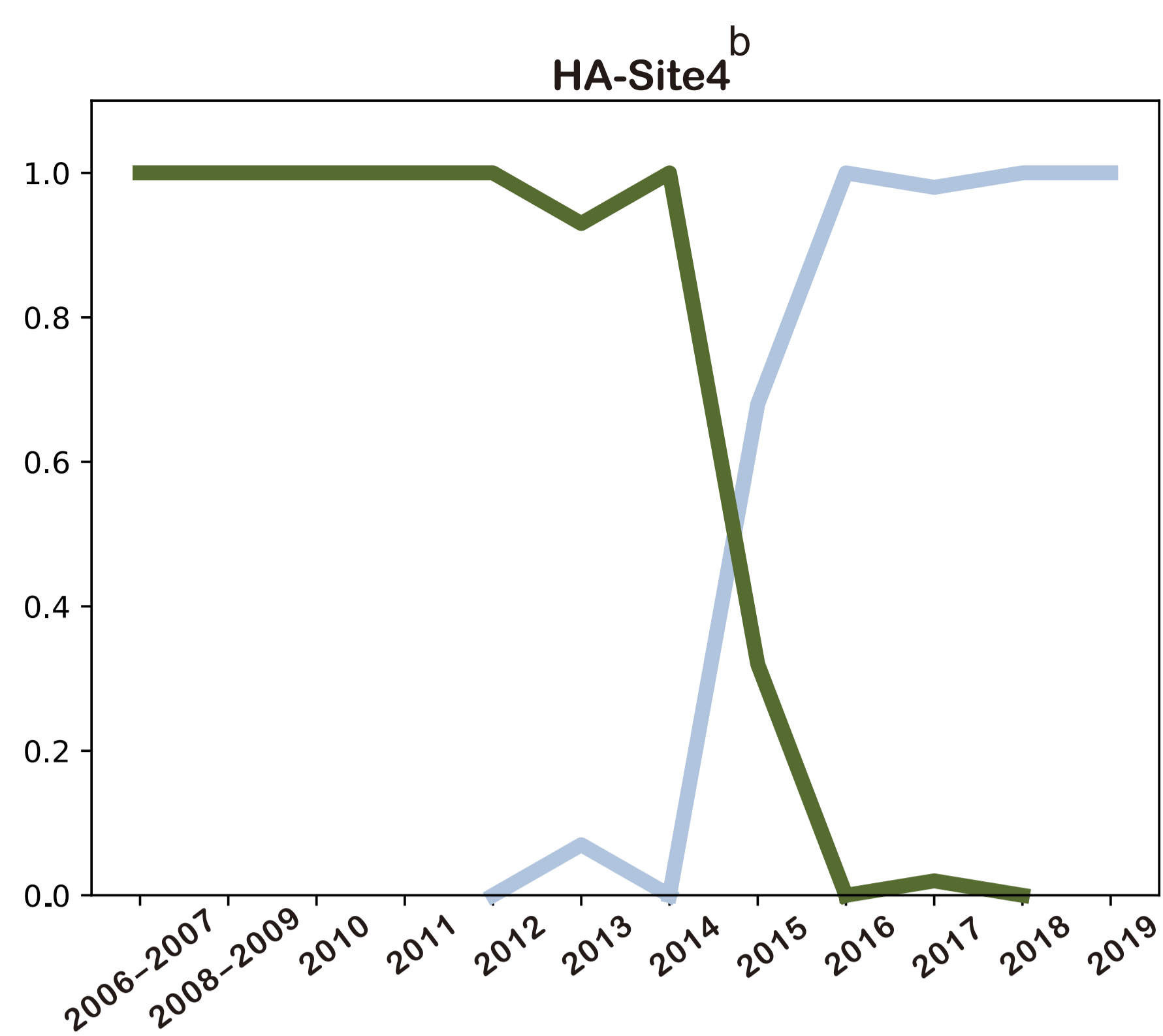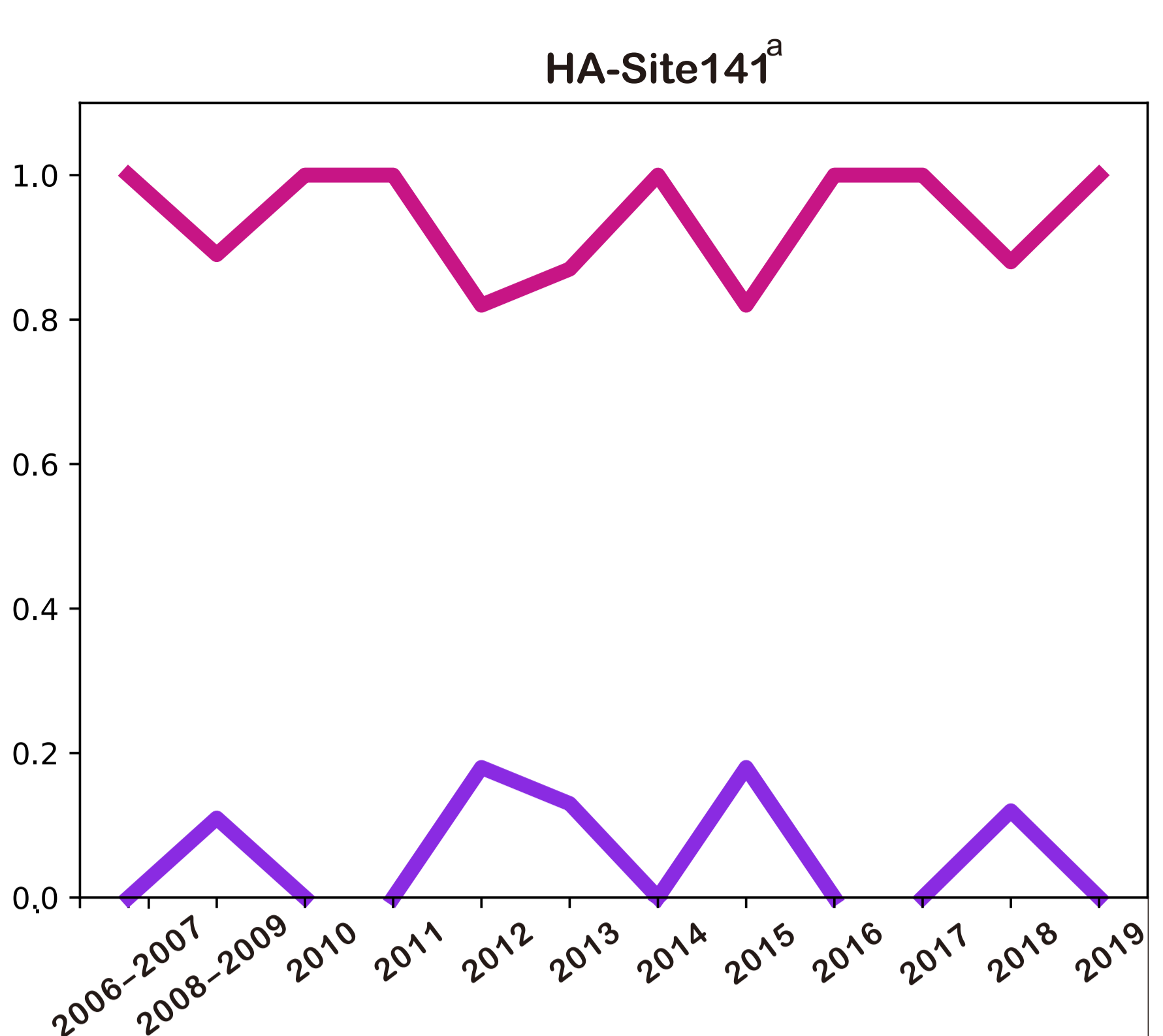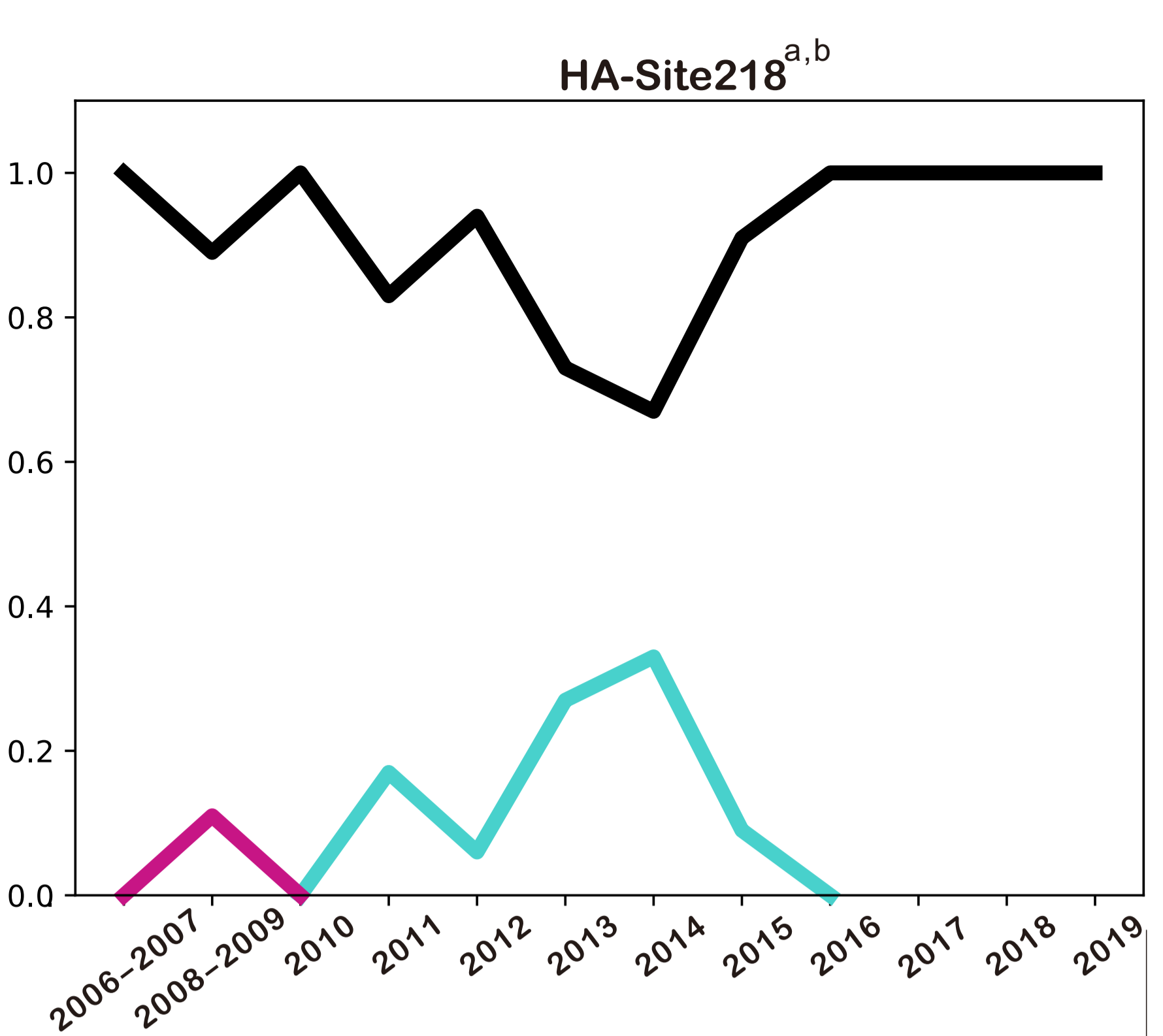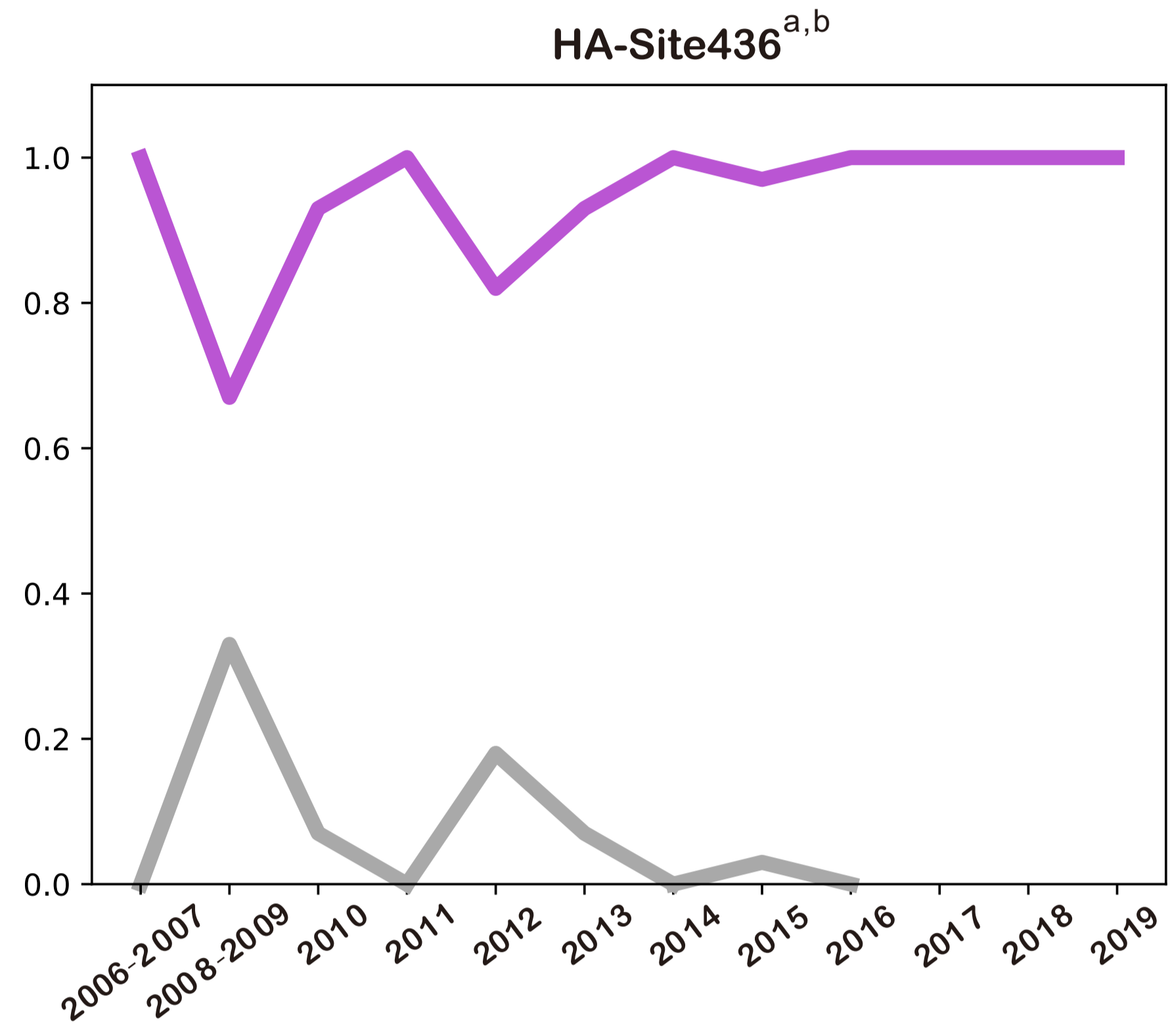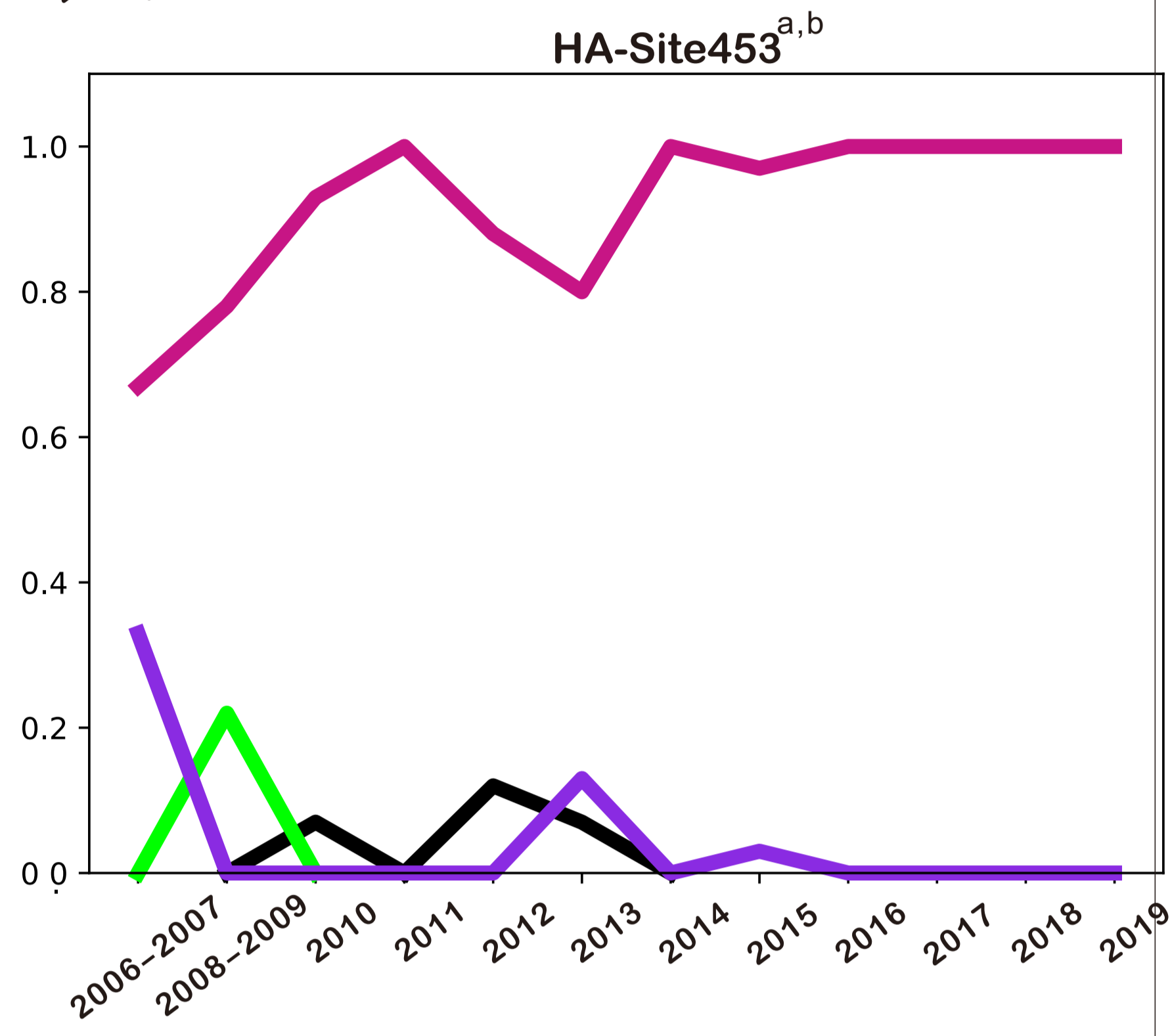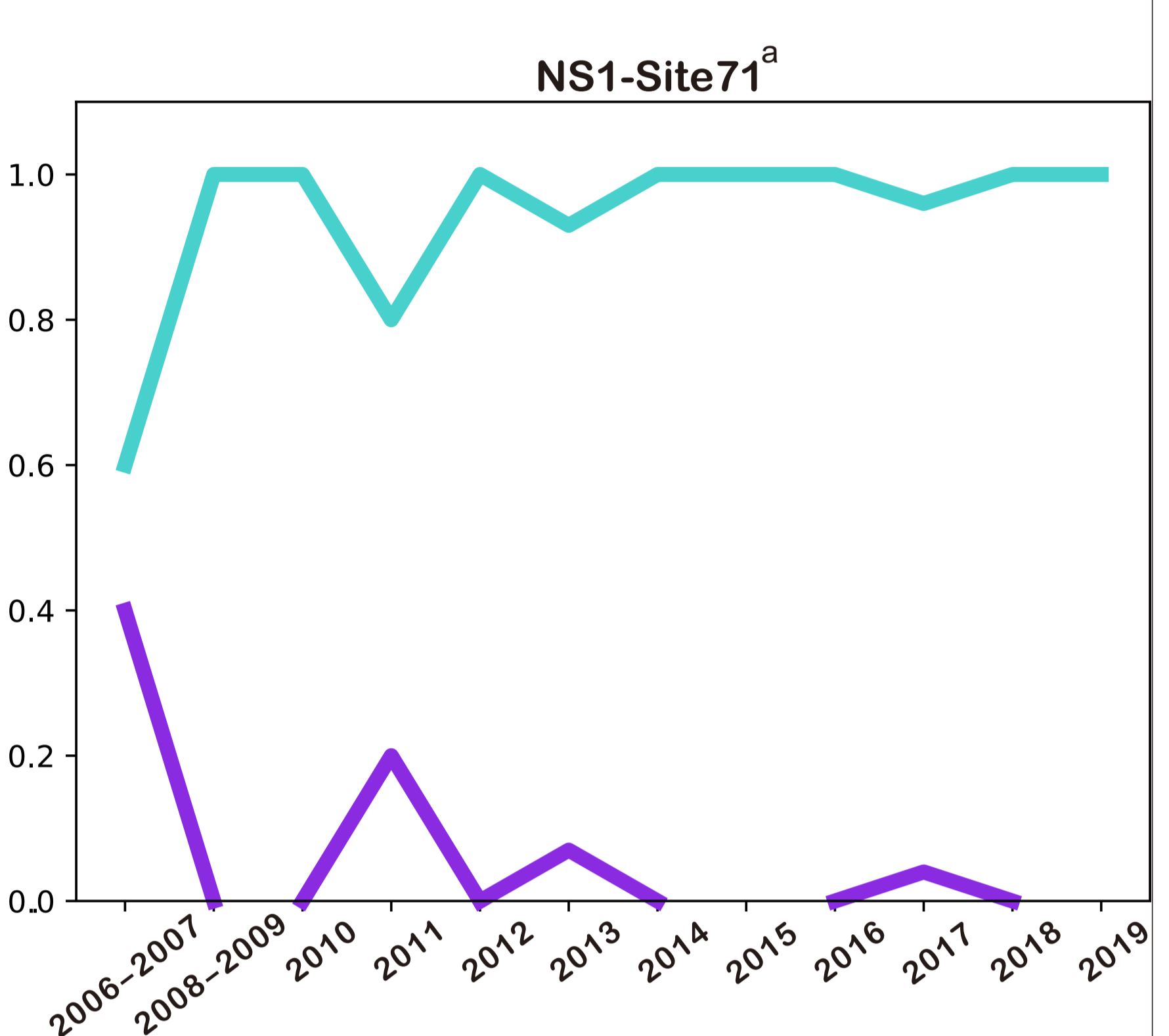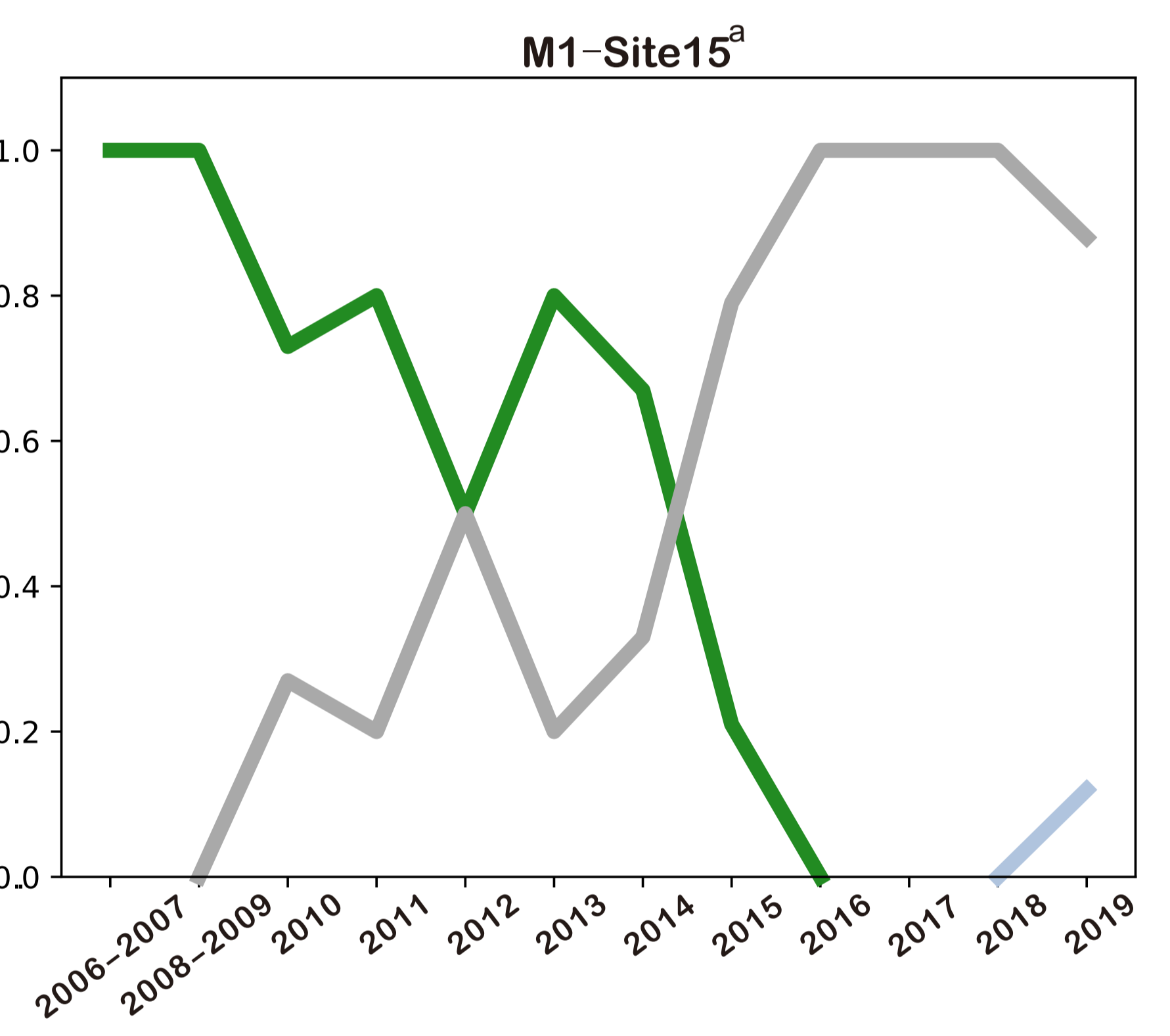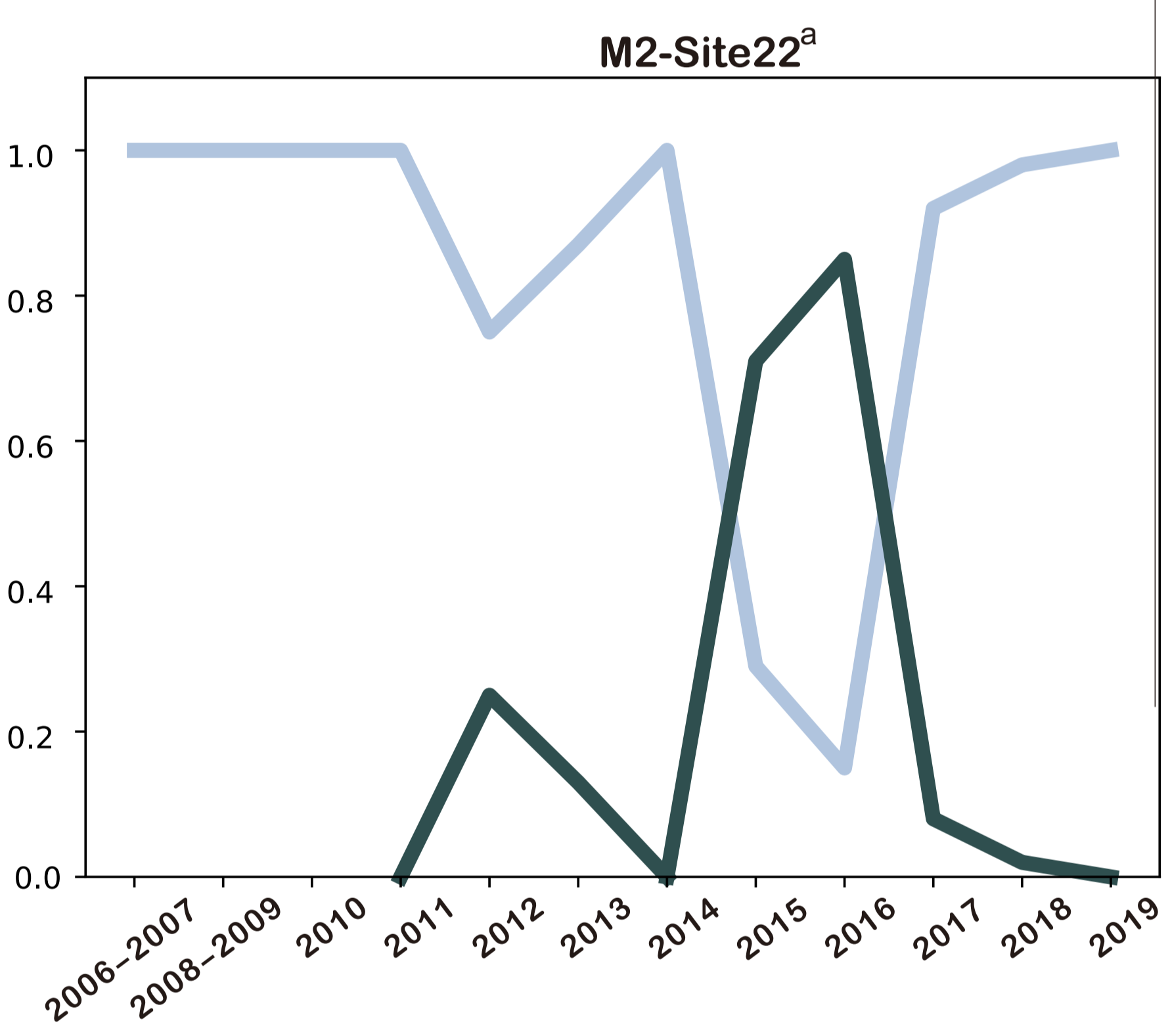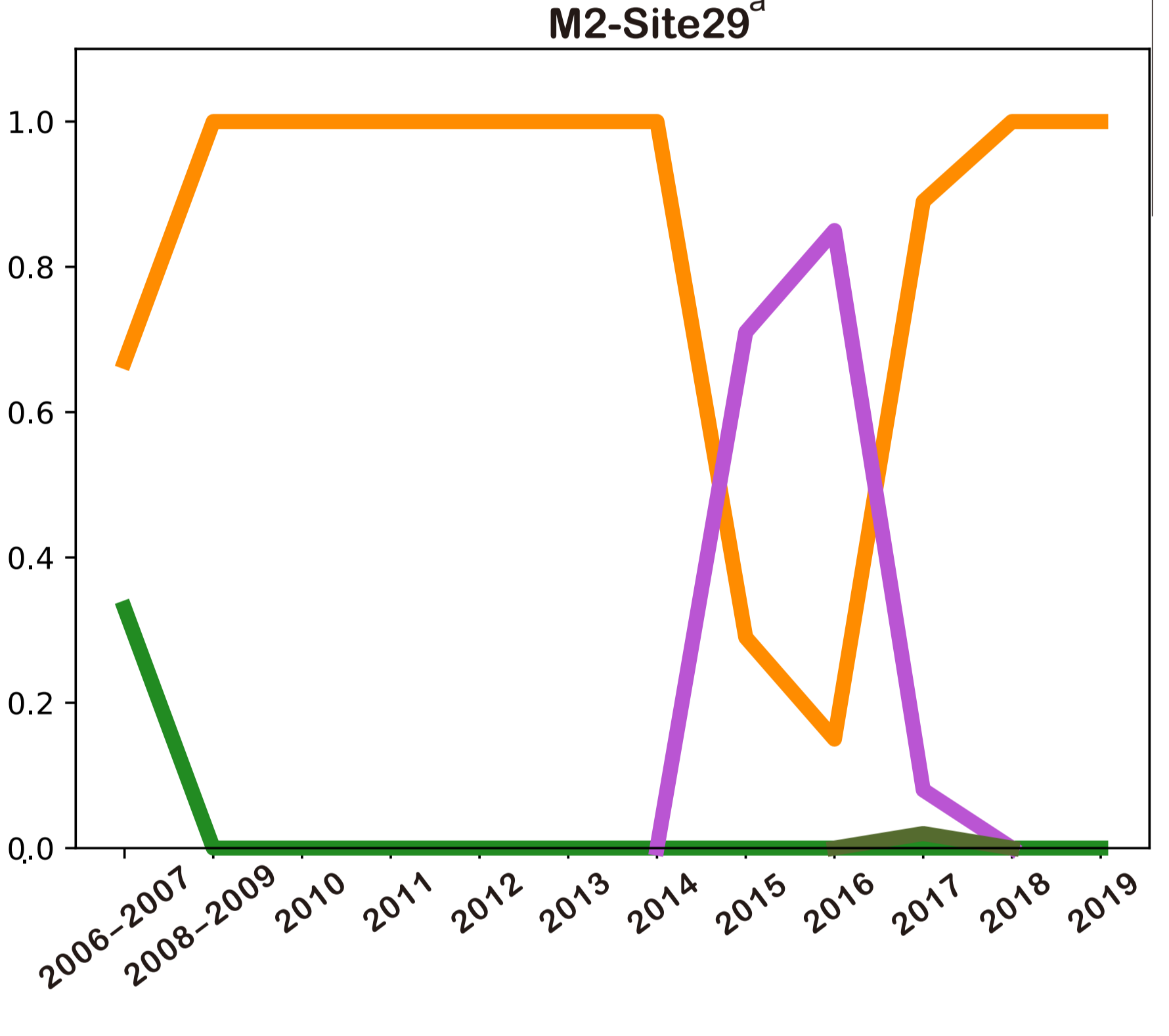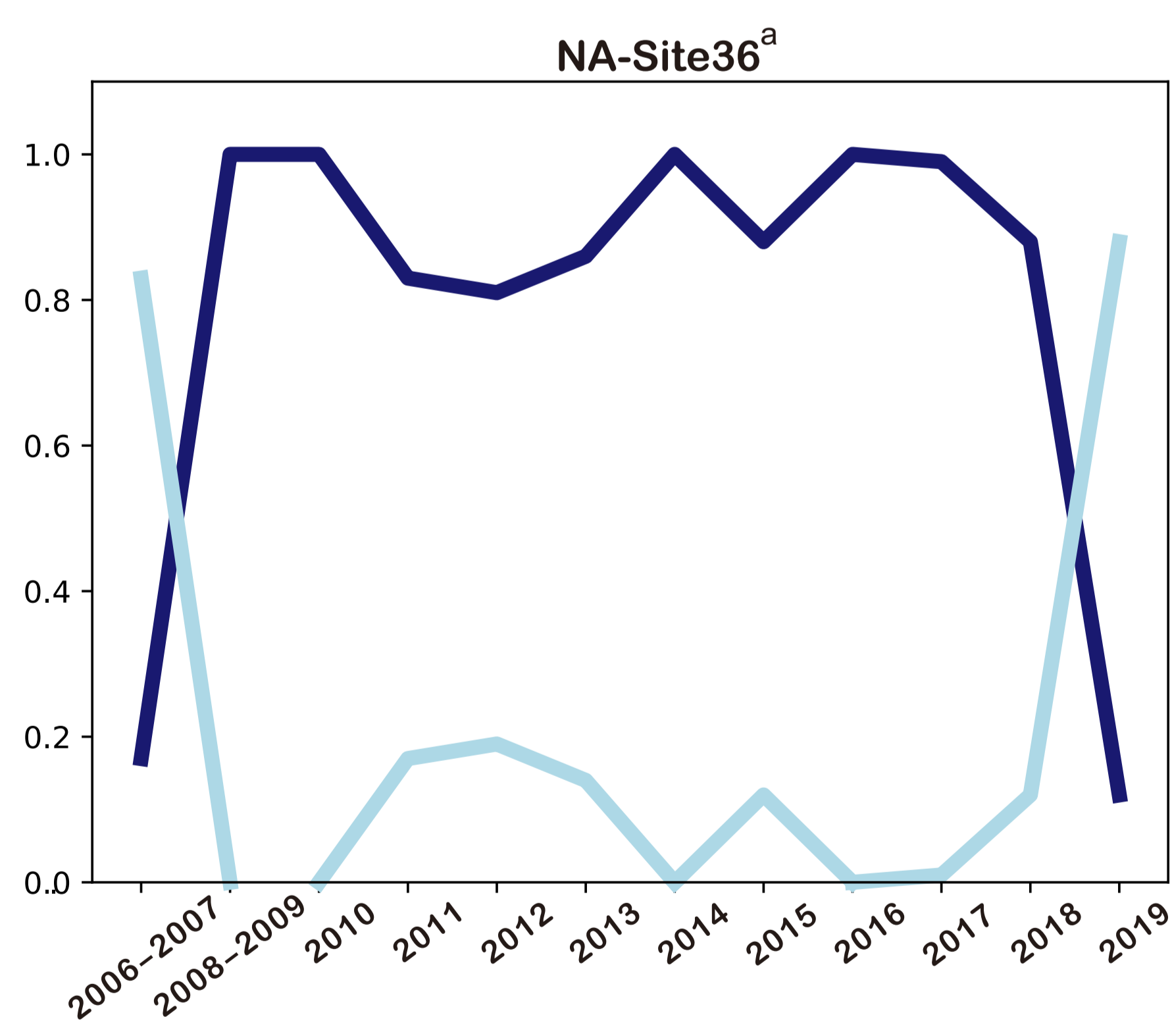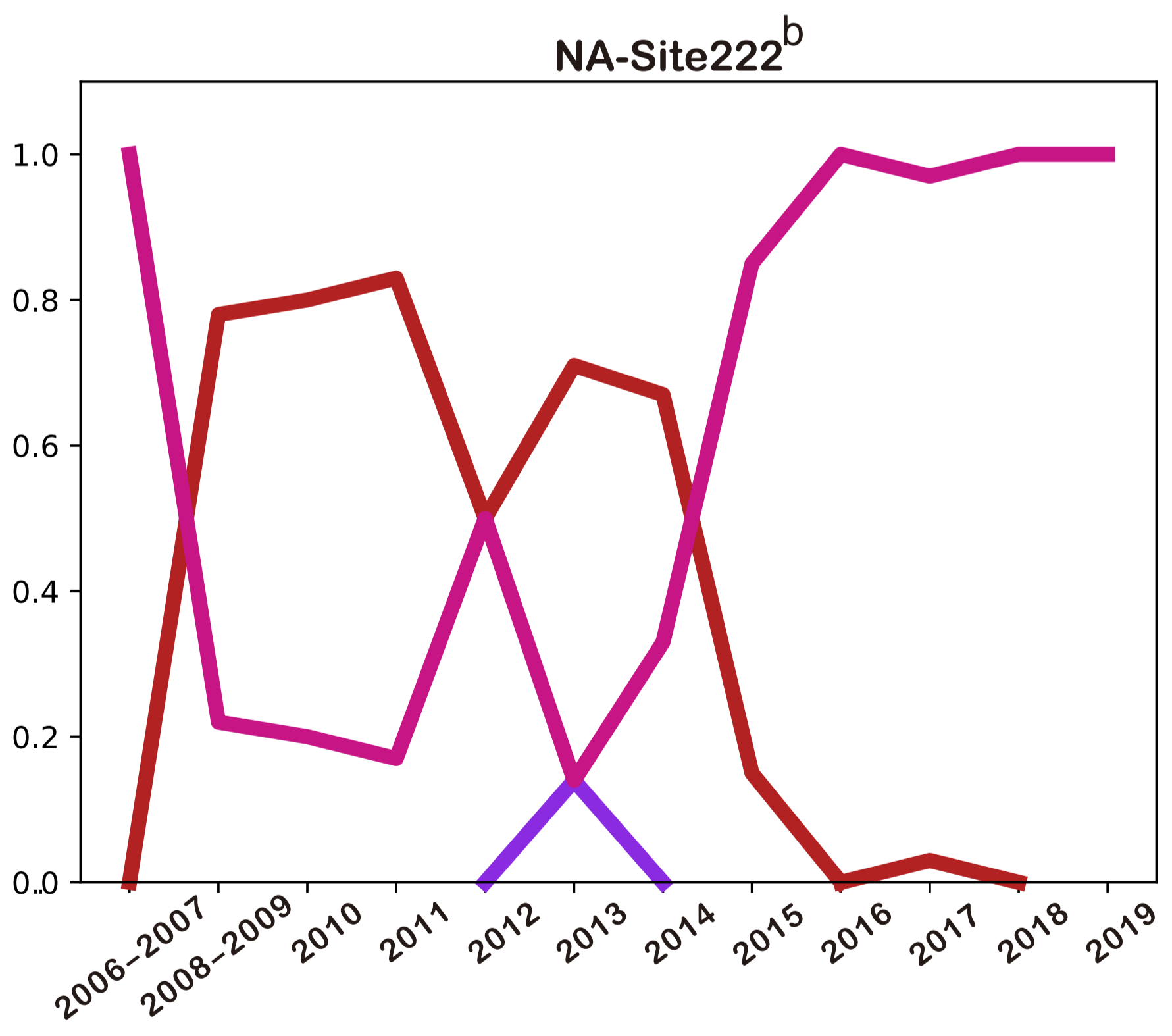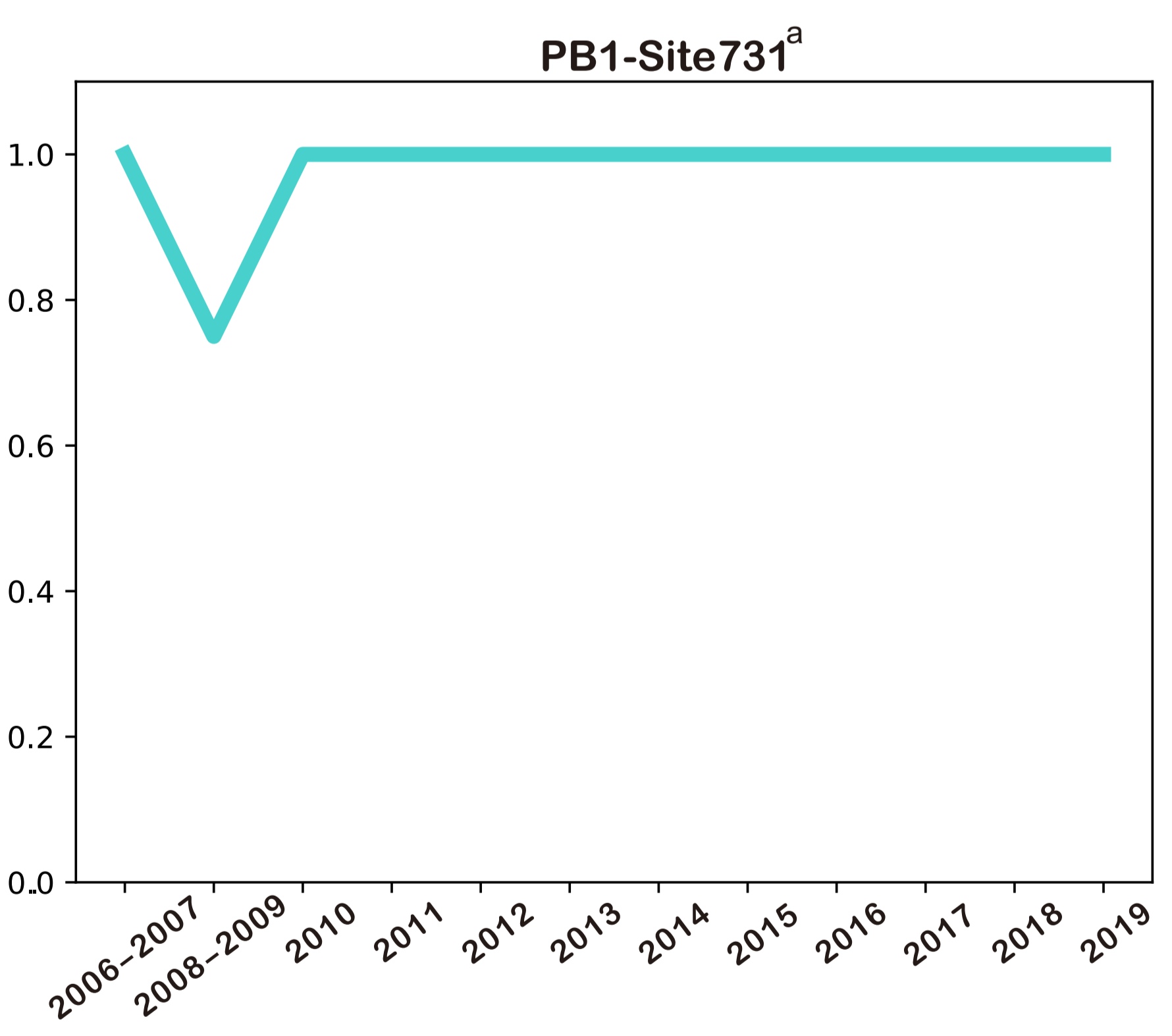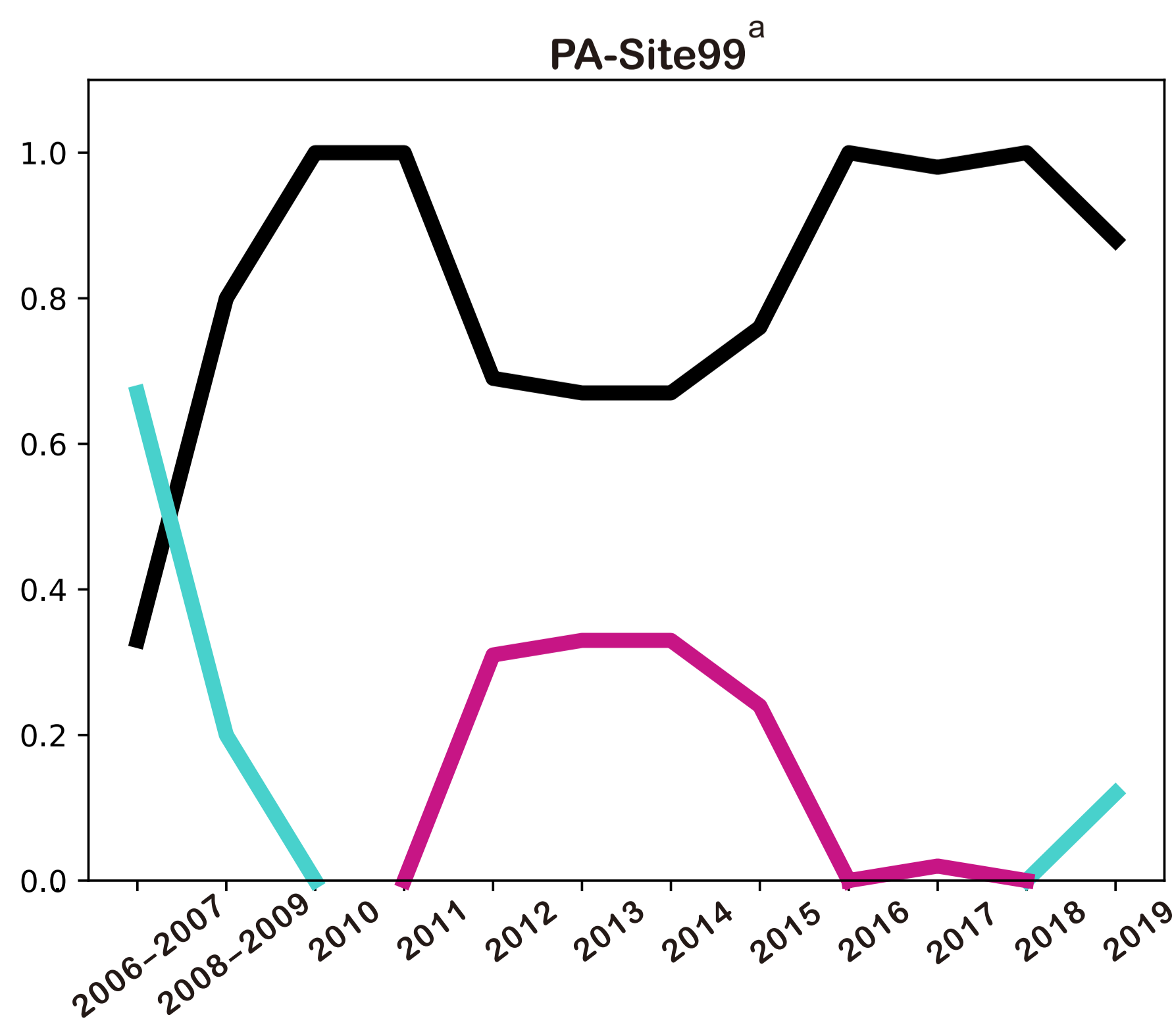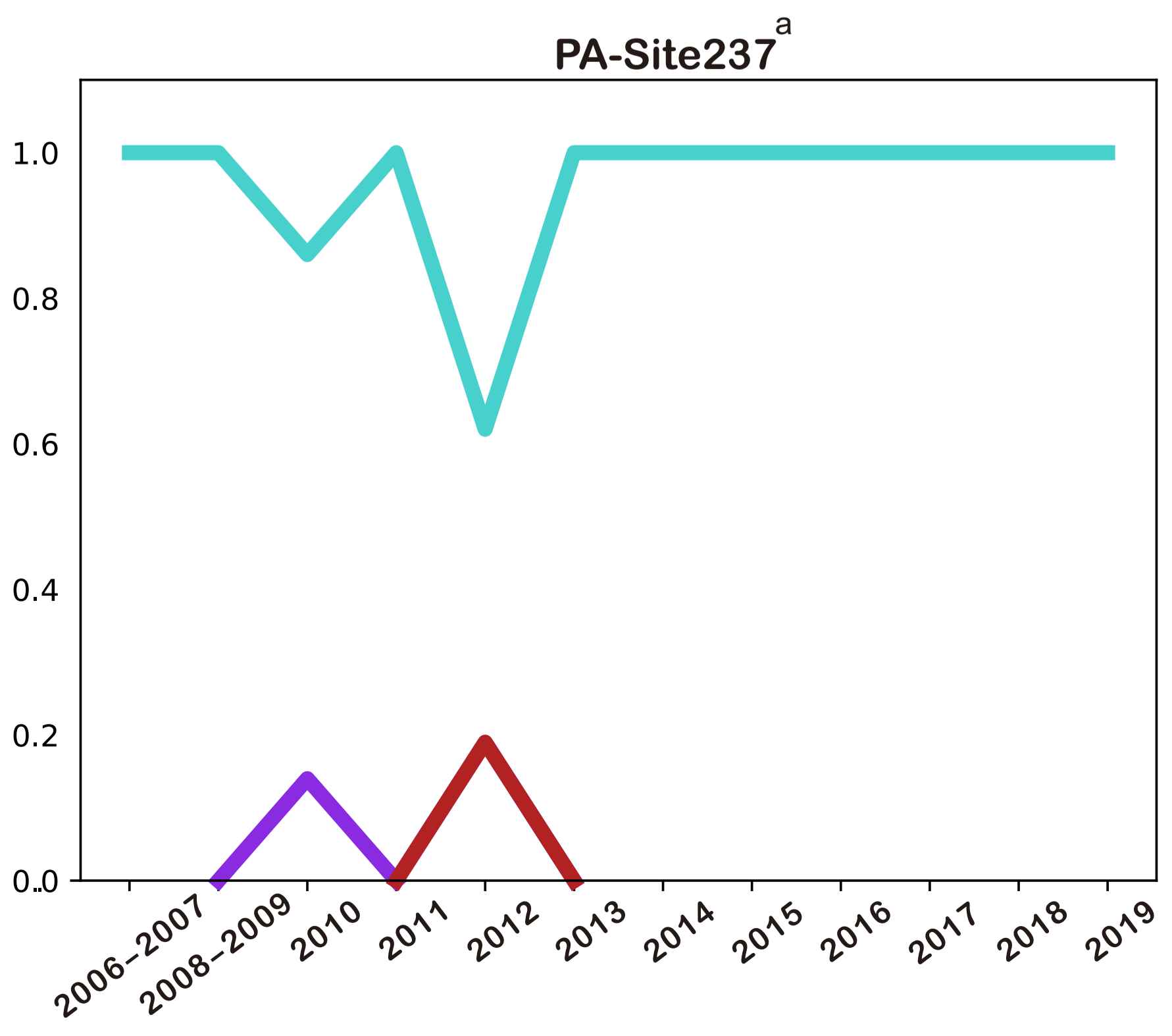

Supplement: Supplementary Figure 5 — Dynamic changes in amino acid frequency for sites under positive selection (dN/dS > 1) during the circulation phase of H3N2 CIV. aPositive selection sites detected in current study. bPositive selection sites detected by He et al. (2019). [file Data_Sheet_5.PDF]
